# N/S element transformation modulating lithospheric microbial communities by single-species manipulation

**DOI:** 10.1186/s40168-023-01553-7

**Published:** 2023-05-16

**Authors:** Shun Yao, Tianzhi Jin, Lu Zhang, Yong Zhang, Rui Chen, Qian Wang, Mingjie Lv, Chuxiao Hu, Ting Ma, Wenjie Xia

**Affiliations:** 1 Key Laboratory of Molecular Microbiology and Technology, Ministry of Education, College of Life Sciences, Nankai University, Tianjin, 300071 People’s Republic of China; 2 Institute of Crop Germplasm and Biotechnology, Tianjin Academy of Agricultural Sciences, Tianjin, 300381 China

## Abstract

**Background:**

The lithospheric microbiome plays a vital role in global biogeochemical cycling, yet their mutual modulation mechanisms remain largely uncharted. Petroleum reservoirs are important lithosphere ecosystems that provide desirable resources for understanding microbial roles in element cycling. However, the strategy and mechanism of modulating indigenous microbial communities for the optimization of community structures and functions are underexplored, despite its significance in energy recovery and environmental remediation.

**Results:**

Here we proposed a novel selective stimulation of indigenous functional microbes by driving nitrogen and sulfur cycling in petroleum reservoirs using injections of an exogenous heterocycle-degrading strain of *Pseudomonas*. We defined such bacteria capable of removing and releasing organically bound sulfur and nitrogen from heterocycles as “bioredox triggers”. High-throughput 16S rRNA amplicon sequencing, metagenomic, and gene transcription-level analyses of extensive production water and sandstone core samples spanning the whole oil production process clarified the microbiome dynamics following the intervention. These efforts demonstrated the feasibility of *in situ* N/S element release and electron acceptor generation during heterocycle degradation, shifting microbiome structures and functions and increasing phylogenetic diversity and genera engaged in sulfur and nitrogen cycling, such as *Desulfovibrio*, *Shewanella*, and *Sulfurospirillum*. The metabolic potentials of sulfur- and nitrogen-cycling processes, particularly dissimilatory sulfate reduction and dissimilatory nitrate reduction, were elevated in reservoir microbiomes. The relative expression of genes involved in sulfate reduction (*dsrA*, *dsrB*) and nitrate reduction (*napA*) was upregulated by 85, 28, and 22 folds, respectively. Field trials showed significant improvements in oil properties, with a decline in asphaltenes and aromatics, hetero-element contents, and viscosity, hence facilitating the effective exploitation of heavy oil.

**Conclusions:**

The interactions between microbiomes and element cycling elucidated in this study will contribute to a better understanding of microbial metabolic involvement in, and response to, biogeochemical processes in the lithosphere. The presented findings demonstrated the immense potential of our microbial modulation strategy for green and enhanced heavy oil recovery.

Video Abstract

**Graphical Abstract:**

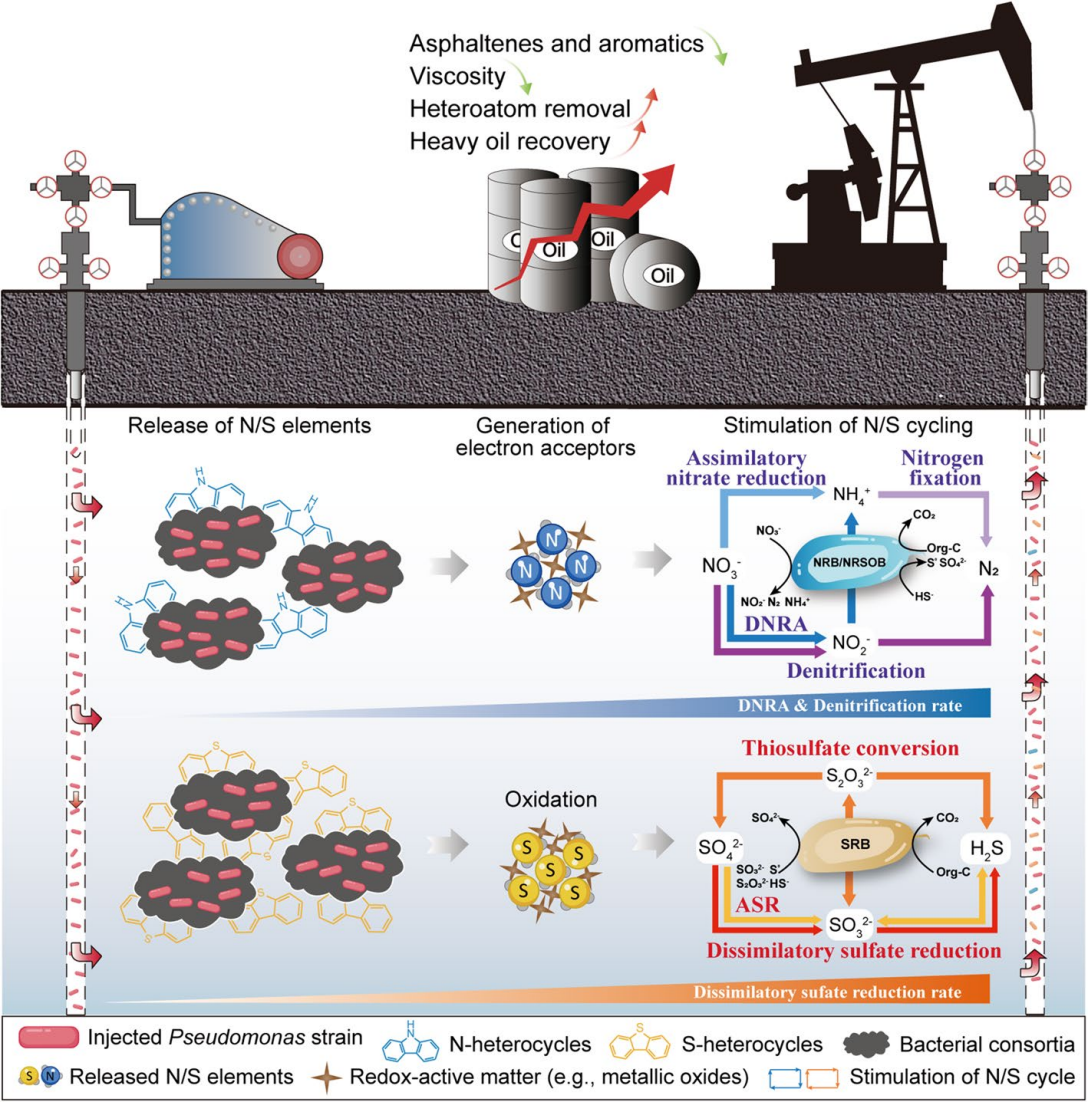

**Supplementary Information:**

The online version contains supplementary material available at 10.1186/s40168-023-01553-7.

## Introduction

Microbial metabolic activities are intricately intertwined with myriads of environmental interactions that collectively shape the biochemical dynamics of ecosystems. The most prevalent and ecologically significant elements, carbon, nitrogen, oxygen, and sulfur, exist in a variety of oxidation states and chemical forms, and their involvements in metabolically driven redox reactions completed by microorganisms substantially influence the biogeochemical cycling of ecosystems [1]. Mounting research on associations between microorganisms and elemental transformations in the habitats of oceans [2–4] and surface soils [5–7] is igniting new interests in the exploration of microbial resources, implications on Earth’s biogeochemistry and global climate, and novel approaches for energy development and environmental remediation [8–10]. However, knowledge about how microbiomes are involved in elemental cycling in lithospheric ecosystems remains limited. Although recent studies have characterized the geographic distribution of microbial life in shallow terrestrial subsurface ecosystems such as groundwater and surficial mangrove sediments [11–13], the modulation and response of the biogeochemical nitrogen and sulfur cycles involving microbial activities in the deep terrestrial lithosphere, particularly petroleum reservoirs that typically occur hundreds of meters below the Earth’s surface, are poorly understood due to difficulties in sample accessibility and processing.

The lithosphere has a complex chemical environment, extreme physiochemical conditions, and phylogenetic and metabolically diverse microbial communities [14]. Despite the relatively low microbial biomass of the lithosphere (2–19% of Earth’s total biomass) [15, 16], the richness of mineral constituents (e.g., Fe, Mn, N, S) and the vast biodiversity of crustal microbiota suggest significant microbial roles and impacts on Earth’s biogeochemical cycling. Petroleum reservoirs, as essential lithosphere components, encompass abundant carbon sources and heteroatomic chemicals in porous minerals. Meanwhile, the tremendous species diversity and metabolic versatility of the microbiome observed in petroleum reservoirs endow them with favorable attributes as natural laboratories for researching microbial participation in lithospheric biogeochemical cycling [17, 18]. Multitudinous functional microorganisms capable of performing redox of carbon, nitrogen, and sulfur compounds have been identified in reservoirs [17, 19]. Multiplex electron flows and elemental transformations will inevitably disrupt microbial homeostasis and induce shifts in the composition, diversity, and metabolic potential of the microbiome when subject to environmental variation. However, interactions between microbial metabolic activities and nutrient cycling remain elusive. Considering that microbial metabolism and growth are predominantly constrained in the nutrient-scarce lithosphere by the availability of electron acceptors [18, 20, 21], utilitarian indigenous microbial enhanced oil recovery (IMEOR) techniques have been proposed adopting the fundamental principle of replenishing reservoir electron acceptors. As promising alternatives to physiochemical approaches, they biostimulate indigenous microorganisms, strengthening microbial metabolism to alter crude oil properties by directly infusing air, nitrate, or sulfate into reservoirs [22]. Nevertheless, injected limited electron acceptors will be indiscriminately utilized and rapidly exhausted during their transportations in subsurface porous rocks; hence, the purpose of selectively activating functional microorganisms for long-term oil production enhancement has not yet been attained.

An enormous challenge in heavy oil recovery is the high-viscosity property resulting from the massive quantities of heterocyclic compounds [23, 24]. These polar compounds incorporate multiple heteroatoms, such as nitrogen, sulfur, oxygen, and metals (e.g., V, Fe, Ni) on their lattices of aromatics [23–25]. Therefore, the removal of heteroatoms is paramount for enhancing oil production (by reducing oil viscosity), alleviating downstream refining operations, and upgrading oil value. In particular, it is anticipated that the release of N/S elements due to carbon-sulfur and carbon-nitrogen bond cleavage could potentially yield electron acceptors (e.g., nitrate and sulfate) by microbial or redox-active matter-mediated oxidation processes [20, 26–30], thereby altering the microbial ecology and further driving the cryptic nitrogen and sulfur cycles in the lithosphere. Microorganisms with superior heterocycle decomposition capabilities will serve as triggers for fueling element cycling and microbial community succession in the subsurface. However, the isolation of robust heterocycle-degrading bacteria that are adaptable to anaerobic environments has remained challenging, and the influence of introducing exogenous heterocycle-degrading bacteria on autochthonous microbial communities and interaction networks of microbial ecology in the lithosphere has yet to be resolved.

Here, we presented an effective microbial regulatory strategy for driving nitrogen and sulfur cycles and stimulating functional microorganisms by introducing exogenous heterocycle-degrading bacteria into heavy oil reservoirs. A facultative anaerobic bacterial strain *Pseudomonas* sp., which had been demonstrated to have remarkable performance in S,N-heterocycle degradation [31, 32], was isolated and charged into heavy oil reservoirs for field trails. The main objective was to explore the microbial succession and involvement in biogeochemical cycling following the modulation of the exogenous bacteria and its application prospect for recovering heavy oil resources. We characterized the consecutive taxonomic and functional profiles of production water (PW) and pristine sandstone core samples over the production lifetime of four oilwells. Through 16S rRNA, metagenomic, and quantitative gene transcription-level analyses, we were able to decipher the key role of the in situ release of N/S elements in structuring the indigenous microbial community of petroleum reservoirs. In addition, we examined the chemical constituents of heavy oil, PW, and gas samples to track the flow of N/S elements in varying valence states, with a particular focus on potential electron acceptors (e.g., nitrate and sulfate).

## Materials and methods

### Geochemical features of pristine petroleum reservoirs

**Fig. 1:**
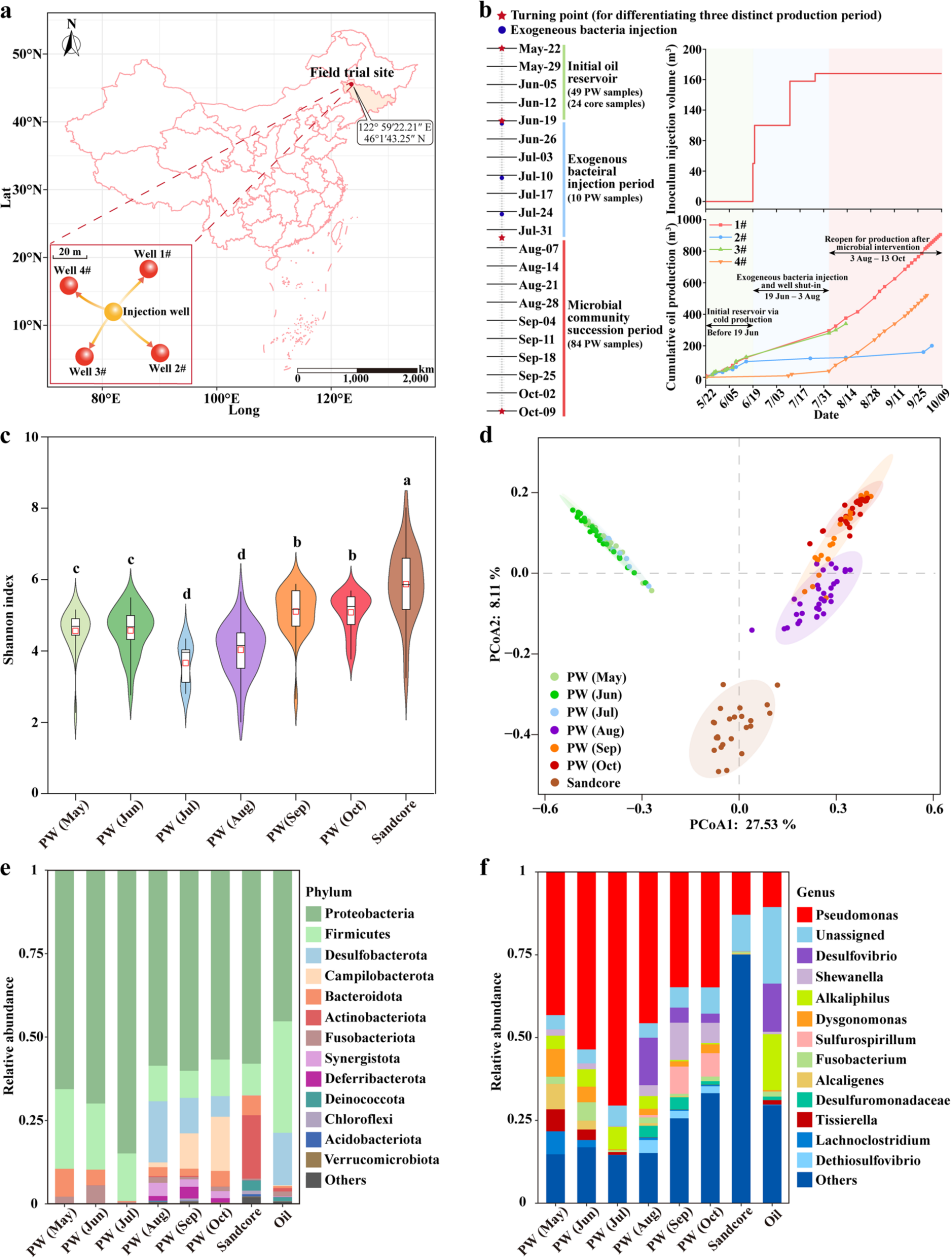
Temporal variations in the microbiota of production water (PW) (*n* = 143), sandstone cores (*n* = 24), and production oil (*n* = 3). **a** Location of the study site and oilwell information of the field experiment, illustrating the operation mode of one-well injection and four-well production. **b** The field trial design, sampling scheme, and oil production details. Initial oil recovery was accomplished by conventional cold approaches, while our microbial modulation strategy was applied by injecting exogenous bacteria in four batches, accompanied by a shut-in period. Cumulative crude oil production was intensively monitored and documented throughout the investigation period. **c** Bacterial α–diversity of production water and sandstone cores was examined by the Shannon index showing the richness and evenness of microbiota. Different letters above violin plots indicate significant differences (Kruskal–Wallis nonparametric rank-sum test, *P* < 0.05). **d** Bacterial β–diversity of principle coordinates analysis (PCoA) based on Bray–Curtis distance. **e** Bacterial composition at the phylum level. **f** Bacterial composition at the genus level

To explore the temporal variations of the lithosphere’s microbiome, we collected extensive microbiome data including 143 production water (PW), 24 sandstone cores (lithospheric minerals), and 3 crude oil samples from four adjacent newly developed wellheads of Zhenlai Oilfield, which are located on the west slope of southern Songliao Basin, China (122.592° E, 46.143° N). The four production wells were distributed in a 50 × 50 m square well pattern, thereby exhibiting similar geochemical characteristics (Fig. 1a). The oil-bearing strata of the reservoir are approximately 200 m deep subterranean and characterized by low temperature (15℃), high porosity (35%) and permeability (2 205 mD), poor cementation (primarily argillaceous with a content of 2–8%), minor diagenesis, low water cut (40–50%), and moderate salinity (8000 mg L^−1^ NaCl). Crude oil recovered from wells has an extraordinarily high viscosity of 1,750,000 mPa·s at reservoir temperature and low API gravity of 9–12 (Supplementary Dataset S1), representing the typical extra heavy oil that is difficult to exploit by conventional production approaches [23].

The oil content test of the cores retrieved from four wells showed a high oil content of the oil sand layer (10.93 ± 0.77%), especially at the bottom of the reservoir (8.53–15.48% at the depth of 196.5–203.7 m; Supplementary Dataset S1). Oil composition was analyzed by the SARA (an acronym for saturates, aromatics, resins, and asphaltenes) method [33], indicating that the pristine oil obtained from wellhead perforation consists of large fractions of asphaltenes (15.31% ± 4.60%) and aromatics (15.55% ± 1.34%). The lithology of sandstone cores reflecting geological structures of local reservoirs was analyzed by measuring whole-rock mineral constituents and elemental concentrations using X-ray diffraction (XRD) and X-ray fluorescence (XRF), respectively. The sandstones were identified as three principle lithologies: quartz (SiO_2_, 51.6%), albite (NaAlSi_3_O_8_, 38.2%), and muscovite (KAl_2_(AlSi_3_O_10_)(OH)_2_, 10.3%). The geochemical composition of sandstones contained 69–77% silica (SiO_2_), 14–17% alumina (Al_2_O_3_), 3–4% potassium oxide (K_2_O), 2–4% soda (Na_2_O), 1–4% iron oxides (Fe_2_O_3_), and other trace elemental oxides. Ion analysis indicated that the natural stores of sulfate (0.07 mg/g) and nitrate (0.01 mg/g) in sandstones are present in exceedingly low concentrations (Supplementary Dataset S1).

### Field trial design and microbial modulation strategy

Petroleum exploitation via conventional cold production approaches had encountered bottlenecks with poor production efficiency since May due to the high viscosity and immobility of heavy oil (Fig. 1b). With the purpose of accelerating oil recovery by anthropogenic manipulation of reservoir microbiomes, the strain *Pseudomonas* WJ-6 (deposited in the China General Microbiological Culture Collection Center with the number of CGMCC4402) was isolated and its morphological, physiological, and phylogenetic characteristics were well elaborated previously [32]. A total of 168 m^3^ of bacterial inoculum containing concentrated exogenous strain WJ-6 (Genebank No. KF155141) was pumped at a rate of 6 m^3^ h^−1^ through a central water injection well into the oil-bearing strata in four batches (between June 19th and July 25th, 2020). The injection inoculum was prepared by mixing harvested dense *Pseudomonas* cells from high-density fermentations with local clean groundwater (Supplementary Methods and Fig. S17). During the period, production wells were shut down periodically, rendering an interval for microbial interaction and hydrocarbon degradation. These four wells were then reopened on August 3rd for enhanced oil production until October 13th. Therefore, the biostimulation process was roughly divided into three stages: (1) the initial reservoir production period (May and June) prior to microbial modulation; (2) the exogenous bacterial injection period (July); and (3) the microbial community succession period (August to October) following the intervention (Fig. 1b). Cumulative oil recovery was recorded every 2 days and the concentration of hydrogen sulfide in reservoirs was monitored using a portable H_2_S detector (GT903-X-H2S).

Production fluids consisting of oil/water mixtures were collected across an extended time spanning from May 22nd to October 13th, while core minerals, perceived as representatives of the pristine undisturbed subsurface biosphere, were retrieved from 185 to 205.5 m below the surface during oil exploration (January, 2020).

### Sample collection, characterization, and DNA extraction

To acquire high-quality consolidated core slices without cracks and contamination, professional geologists were invited to assist us in drilling and sampling with oil-based drilling mud and coring barrels prior to oil extraction [34]. The complete procedures for core sampling and processing were outlined in Supplementary Methods and Fig. S1. Briefly, fresh core sections experienced a series of on-site pretreatments, obtaining multiple core slices approximately 10–30 cm in length. They were immediately sealed in two-layer sterile vacuum tinfoil containers and clearly labeled with geological information before being transported with dry ice to laboratories. Once in the lab, a portion of the inner core slices was handled for downstream geochemical analysis and DNA extraction by aseptically crushing the core slices using a sterile rock hammer and bolster chisel and further grinding them into powders with a sterile mortar and pestle. The remainder was stored at −20°C for backup. Production fluid samples collected at wellheads were filled to the top of 500 mL sterilized boro glass bottles with screw caps to prevent oxygen intrusion and contamination (see Supplementary Methods). The oil-water mixture samples, labeled with exact sampling time and well number, were promptly transported to the laboratory for compositional and microbiological analyses. After isolating the water layer from the mixture by decantation, the microbial cells in the water layer were harvested using centrifugation at 4°C and 13,000×*g* for 10 min in a high-speed centrifuge (Beckman, USA). The cell deposits were used for later DNA extraction, while the supernatant was collected and filtered through 0.22-μm woven polyethersulfone membranes for inorganic anion detection using ion chromatography (Thermo Fisher, ICS-1100), equipped with a new Dionex IonPac™ AS14 Carbon Eluent Anion-Exchange Column. Details regarding the sampling information and geochemical analyses are summarized in Supplementary Dataset S1.

Total genomic DNA from PW was extracted using a Mag-Bind® Environmental DNA Kit (Omega Bio-Tek, USA), whereas the DNA extraction of sandstone cores is more challenging due to its low biomass and absorption of nucleic acids to the charged clay surfaces, thereby applying an improved DNA extraction protocol using PowerSoil DNA isolation kit (Mo Bio Laboratories, Carlsbad, USA) following the manufacturer’s instructions with modifications as described in Supplementary Methods. Strategies for minimizing contamination are critical in microbiome studies, especially for treating low-biomass samples. We included two types of negative controls: DNA extraction blank controls and no-template amplification controls, in accordance with the “RIDE” minimum guideline checklist [35]. They were processed alongside biological samples and sent for library preparation and sequencing to assess the potential contaminant taxa from extraction kits, reagents, and laboratory environments (Supplementary Results).

### Enumeration of bacterial density in sandstone cores and PW

To ensure an accurate estimation of the absolute cell population in petroleum reservoirs, we used both real-time fluorescent quantitative PCR (qPCR) and culture-based methods to measure the cell density of total bacteria and culturable aerobic and anaerobic bacteria, respectively (Supplementary Methods). Bacterial 16S rRNA genes were amplified using primers 27F/338R on the platform of Bio-Rad MYiQ2 real-time fluorescent quantitative PCR. The qPCR results from triplicate samples were calculated from the standard curve and expressed in colony-forming units (CFU) per mL of PW (Supplementary Results and Table S5). Cell populations of culturable bacteria were determined via plate counts as previously described [36, 37], with several modifications. Two grams of pulverized core powders was prepared into 20-mL core suspensions, which were plated on R2A agar plates after appropriate serial dilutions. Aerobes and anaerobes were enumerated separately after 5 and 21 days of incubation at 30℃ under oxic and anoxic conditions.

### Gene expression abundance analysis by reverse transcription real-time quantitative PCR (RT-qPCR)

Enough bacterial cells were collected from a vast volume of fresh PW samples (80 mL) by centrifugation at 4500 rpm for 8 min, considering the low bacterial cell density. Bacteria was immediately resuspended by adding 1 mL Trizol to avoid RNA degradation. Total RNA was extracted using a RNAprep Pure Cell/Bacterial Kit (TIANGEN) according to the manufacturer’s protocol. The RNA template was qualitatively assessed and quantified using a NanoDrop 2000 spectrophotometer (Thermo Scientific, Waltham, MA, USA). Approximately 1 μg extracted RNA was reverse transcribed into cDNA at 37℃ for 5 min, 50℃ for 30 min, and 85℃ for 5 min using reverse transcriptase (StarScript III All-in-one RT mix with gDNA remover, GenStar).

RT-qPCR was performed on the platform of Bio-Rad MYiQ2 real-time fluorescent quantitative PCR targeting core N, S cycling marker genes, including *napA* (encoding the periplasmic nitrate reductase responsible for catalyzing the first step of denitrification), *nosZ* (encoding the nitrous oxide reductase catalyzing the last step of denitrification), *dsrA* and *dsrB* (encoding the alpha- and beta-subunits of dissimilatory sulfite reductase). Each reaction contained a 20 μL mixture with 40 ng cDNA, 0.4 μL of each primer (20 pmol), and 10 μL of 2×SYBR RealStar Green Fast qPCR Mixture (GenStar). Details of thermal cycling parameters and primer sequences for each gene are listed in Supplementary Table S1. All reactions were conducted in triplicate with no-template controls in each run. Genes’ relative expression abundance was quantified by 2^−ΔΔCT^ method [38], using 16S rRNA as an internal reference gene. The fold-change in gene relative expression was calculated by dividing the expression level by the averaged gene expression value of the initial reservoir samples.

### 16S rRNA sequencing and processing

The V4–V5 regions of bacterial 16S rRNA were amplified using the universal primer pair 515F (5′-GTGCCAGCMGCCGCGGTAA-3′) and 907R (5′-CCGTCAATTCCTTTGAGTTT-3′). PCR amplicons were sequenced on the Illumina Novaseq 6000 platform (Novogene Co., Beijing, China). The obtained sequence reads were processed using QIIME v2-2021.2 following the official tutorial [39]. In brief, after quality control by removing low-quality sequences (quality score < 25), the remaining raw reads were denoised and clustered into exact amplicon sequence variants (ASVs), which represent 100% operational taxonomic units (OTUs), using DADA2 [40]. To rule out spurious features, low-abundance ASVs ($n$ ≤ 5) were filtered out. The rarefied ASV tables were resampled to the number of the least sequence of samples ($n$ = 16,000 when all environmental samples were considered; $n$ = 25,000 when PW samples were exclusively considered), which were qualified for downstream analysis.

### Metagenomic sequencing and processing

To profile changes in gene abundance and metabolic potentials of the PW microbiome during the entire modulation process, PW samples from various stages with prominent features were chosen for metagenomic sequencing. Deep sequencing libraries were constructed and sequenced on the Illumina NovaSeq 6000 platform using a paired-end 150-bp strategy. Metagenomic sequencing finally generated 41.19 Gb of clean reads. The trimming procedure and removal of host contaminations were performed using Trimmomatic v0.39 [41] and Bowtie2 v2.4.2 [42], respectively. The quality of reads was repeatedly checked by FastQC v0.11.9 [43]. The trimmed reads were assembled by MEGAHIT v1.2.9 [44] and the assembled contigs were annotated by Prokka v1.14.6 [45]. The predicted genes from all samples were clustered using CD-HIT v4.8.1 [46] to establish a non-redundant gene set based on 95% similarity. Gene relative abundance was quantified by salmon v1.3.0 [47]. Non-redundant genes were mapped against the Kyoto Encyclopedia of Genes and Genomes (KEGG) database [48] for functional annotations using eggNOG-mapper v2.0 [49]. Genes with KO annotations involved in target metabolic pathways were selected for functional analysis.

Contigs from individual samples were separately clustered into bins to recover near-complete genomes using the MetaWRAP [50], which integrates multiple binners of MaxBin2 [51] and MetaBAT2 [52] and performs better results than using a single binner through our preliminary tests. The obtained bins were further dereplicated and refined using dRep [53], with cut-off settings of 90% whole-genome average nucleotide identity (ANI) clustering, and an overall metagenome-assembled genome (MAG) completeness of ≥50% and contamination of ≤10%. Recovered MAGs were taxonomically annotated using the Genome Taxonomy Database (GTDB) Toolkit (v.1.3.0) against the GTDB (release 95) [54, 55]. The phylogenetic tree of MAGs was visualized using iTOL v5 [56].

### Data analysis

#### Microbial community composition and diversity

16S rRNA representative sequences were aligned to the Silva-138.1 database as a reference [57]. We used the Shannon index to represent the bacterial α–diversity calculated using the *diversity* function in QIIME. Bacterial β–diversity was assessed through principle coordinate analysis (PCoA) based on the Bray–Curtis distance of the normalized ASV table using the *vegan* package in R v4.1.2. The Venn network diagram, which shows the linkages and belongings of ASVs among groups, was generated in Cytoscape 3.9.1 [58]. Different sample groups and the involved ASVs were regarded as source nodes and target nodes, respectively. The connectivity and node information was organized using R. Transformation-based redundancy analysis (tb-RDA) was performed using the *vegan* package in R to elucidate how bacterial community dynamics were linked to electron acceptors (${\mathrm{NO}}_{3}^{-}$ and ${\mathrm{SO}}_{4}^{2-}$).

#### Co-occurrence network analysis

The objective of co-occurrence network analysis was to investigate the microbiome complexity and interactions as well as linkages between microbial species and environmental factors. Network topology, such as the clustering coefficient, modularity, degree, and centrality reflects the connectedness of networks and the importance of species [59–61].

To avoid sparse ASVs affecting correlation analyses, 578 ASVs from all samples were involved in the microbe–microbe interaction assessment after filtering out ASVs with a relative abundance <0.01%. In addition, 404 ASVs from the PW samples with relative abundance >0.01% were selected to implement a microbe–environmental factor interaction analysis. The correlation degree was determined using the *psych* package in R. Only strong correlations with Spearman’s correlation coefficients $|r|$ > 0.7 and *p*-value < 0.01 were considered statistically significant. The co-occurrence network between microbes was constructed and visualized using the interactive platform Gephi v0.9.2 [62]. Topological parameters of networks and node-level features were calculated for subnetworks using the *igraph* packages in R [63], including the number of nodes and edges, average degree (the number of connections), diameter, betweenness (the number of paths through a node), clustering coefficients, and modularity. The correlation network graph of microbe–environmental factor interactions was generated in Cytoscape, offering simultaneous insights into negative or positive correlations, ASV relative abundance, and correlation degree.

#### Potential function prediction

To acquire a detailed overview of the universal roles of bacterial populations in PW, the Functional Annotation of Prokaryotic Taxa (FAPROTAX) database (http://www.loucalab.com/archive/FAPROTAX/), which has been extensively applied in predicting metabolic and ecological functions of environmental samples involved in biogeochemical cycling (especially for element cycling), was referred to generate potential functional profiles of all PW samples based on taxa [64]. Moreover, metagenomics analysis was employed to directly estimate functions based on community gene content. Protein sequences of assembled contigs were mapped against the KEGG database using eggNOG-mapper v2. The annotated gene abundance was normalized for comparative analysis.

### Statistical analysis

Taxa with an abundance greater than 0.1% that are significantly different among habitats were identified by Linear Discriminant Analysis Effect size (LEfSe) with default settings [65]. The features of taxonomic and functional profiles that significantly differed between pairs of groups were determined and visualized using STAMP software [66]. The statistical test was based on Weltch’s *t*-test with a *p-*value < 0.05, and Benjamini–Hochberg FDR was applied for test correction. The significant difference in α–diversity was examined by the Kruskal–Wallis nonparametric rank-sum test with an adjusted *p*-value < 0.05 using SPSS v26 statistical software (SPSS Inc., USA). To further assess the relevance among dominant taxa and their correlations with nitrate and sulfate across the succession, Spearman correlations of taxa versus taxa and taxa versus environmental variables were calculated with 95% confidence intervals using the *corrplot* packages in R. SARA content variations were evaluated by a *t*-test at a significance level of 0.05 and expressed as mean ± standard error (SE).

## Results

### Temporal variations in bacterial taxonomic composition and diversity

We generated bacterial taxonomic profiles for individual samples via high-throughput sequencing of 16S rRNA gene amplicons (Supplementary Figs. S3, S4), and a total of 8,108,477 reads were gathered across all PW, lithospheric minerals, and crude oil samples, which were clustered into 16,558 amplicon sequence variants (ASVs). After quality control and filtration, 8432 ASVs were retained for subsequent analysis. The composition of the bacterial microbiota indicated that *Proteobacteria*, *Firmicutes*, and *Bacteroidota* were the dominant phyla across all samples, accounting for over 70% of the total sequences (Fig. 1e, f), which is in accordance with previous reports on petroleum reservoirs [67]. *Pseudomonas* was the most abundant genus in PW (36.6–70.8%) throughout the study period, followed by *Desulfovibrio* (0–14.9%), and *Shewanella* (0.1–12%), which became numerically substantial components of PW microbiota after July. The modulation of exogenous bacteria led to a pronounced bacterial community succession. Specifically, a growing number of taxa using sulfur or nitrogen as electron donors (in the form of sulfide, elemental sulfur, thiosulfate, ammonia, or nitric oxide) or acceptors (sulfate, sulfite, thiosulfate, nitrate, or nitrite) appeared, such as *Desulfovibrio* (phylum *Desulfobacterota*), *Dethiosulfovibrio* (phylum *Synergistota*), *Desulfuromonas* (family *Desulfuromonadaceae*), *Sulfurospirillum* (phylum *Campilobacterota*), *Wolinella* (phylum *Campilobacterota*), and *Shewanella* (phylum *Proteobacteria*). Nevertheless, the abundance of these bacteria associated with nitrogen and sulfur cycling subsequently exhibited distinct trends. The abundance of sulfate- or sulfite-reducing bacteria (SRB, such as *Desulfovibrio*, *Desulfuromonas*, and *Dethiosulfovibrio*) declined steadily after reaching the maximum in August, whereas nitrate- or nitrite-reducing bacteria (NRB, such as *Sulfurospirillum*, *Shewanella*, and *Wollinella* [18]) and several autotrophic nitrate reducing–sulfide oxidizing bacteria (NR-SOB, such as *Sulfurospirillum* and *Pseudomonas* sp. C27), which are capable of coupling sulfide oxidation to nitrate reduction [68, 69], progressively dominated. Contemporaneous measurements of inorganic ions in PW observed that the sulfate concentration had increased rapidly since the end of July and then gradually dropped after peaking on 7th August (6 g/L) (Supplementary Fig. S2b). Ultimately, sulfate content fluctuated at a low level (1 g/L) during September and October. In contrast, the nitrate started to accumulate in late August (Supplementary Fig. S2c). The concentration changes in sulfate and nitrate are basically consistent with the dynamics of SRB and NRB, suggesting the in situ regeneration of electron acceptors and their driving effects on reservoir microbial community succession. In addition to time-scale variations, lithospheric minerals harbored different microbiota from subterranean fluids, manifested in a notably higher relative abundance of *Actinobacteria* (19.1%), which are the primary filamentous microbial colonizers that thrive on the surface or interior of rocks by hyphal attachment to facilitate bacterial protection, biofilm adherence, and mineral acquisitions [70, 71].

Shannon diversity index was used to evaluate the microbiota diversity of richness and evenness, with lithospheric minerals > PW (September and October) > PW (May and June) > PW (August) > PW (July) (Fig. 1c). The microbiota of lithospheric minerals was significantly more diverse than that of PW at each stage, and the microbiota of PW (7, 8, 9, 10) presented a pronounced upward trend in diversity over time (*P <* 0.05, Kruskal–Wallis rank-sum test). Dissimilarities in microbial community structure were clearly distinguished by PCoA based on Bray–Curtis distance (Fig. 1d), which showed that the microbiota from PW and lithospheric mineral samples were divided into three distinct clusters. The microbiota of PW samples before and after intervention were entirely different (PW5, 6, 7 versus PW8, 9, 10) that were separated along the first coordinate axis. In addition, PW samples after being intervened also presented a slight shift from August to October along the second PCoA axis. As expected, the microbiota of lithospheric minerals differed substantially from that of PW owing to the different types of samples and biotopes.

**Fig. 2:**
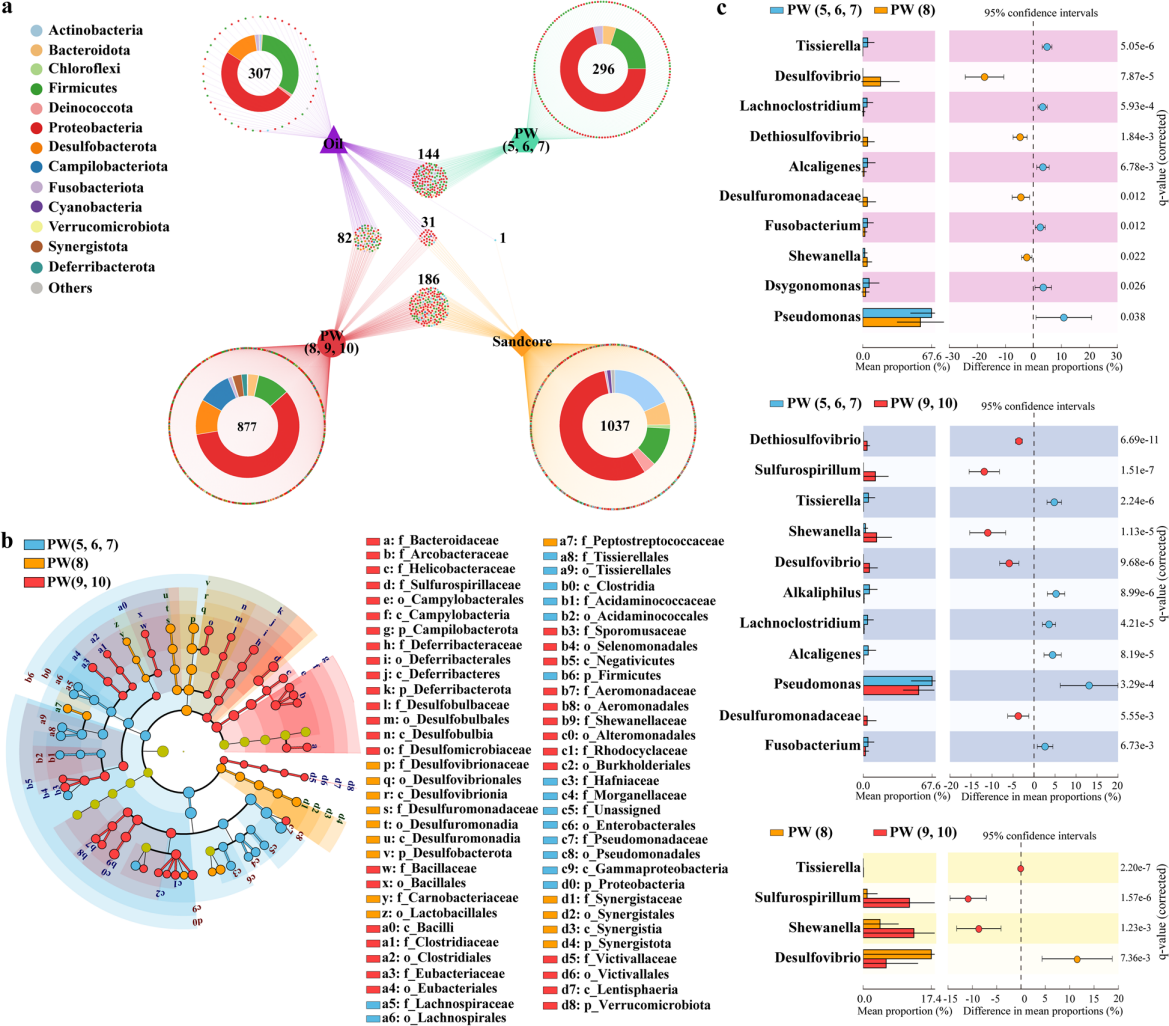
Taxonomic characteristics and linkages among various stages of PW, sandstone core, and oil microbiota. **a** A Venn network graph highlighting both the number and membership of overlapping ASVs detected among various stages of PW, sandstone core, and oil samples. The Venn graph was constructed using a total of 2038 ASVs with a relative abundance of greater than 0.001%. Filled blocks of different shapes and colors represent a collection of samples from specific habitats. Each filled dot denotes an individual ASV. Connections between blocks and dots indicate the belongings of ASVs. **b** Cladogram showing the significantly different taxa among various stages of PW at class, order, and family levels identified by LEFSe analysis (LDA score > 3). **c** Pairwise comparisons of significantly different genera among various stages of PW (adjusted *q-*value < 0.05). The *q-*value is based on Weltch’s *t*-test corrected by Benjamini–Hochberg FDR

We next examined the linkages among groups of samples from various biotopes using a Venn network graph (Fig. 2a), visually presenting both the quantities and membership of shared intersections [72]. A total of 2 038 ASVs with a relative abundance of > 0.001% were identified, and only 31 shared ASVs were found among the three habitats of PW (8, 9, 10), sandstone cores, and oil (Fig. 2a). PW (8, 9, 10) (877 ASVs) and sandstone cores (1 037 ASVs) harbored considerably more diverse species composition than PW (5, 6, 7) (296 ASVs). Intriguingly, the Venn network graph showed no shared ASVs between the two stages of PW, indicating that the bacterial community of PW had been strongly disturbed and changed. PW (8, 9, 10) contained a notable overlap of ASVs with both cores and oil, belonging to many dominant phyla, such as *Proteobacteria*, *Firmicutes*, and *Desulfobacterota* (Fig. 2a). In contrast, the bacterial community composition of PW (5, 6, 7) was highly heterogeneous when compared to other habitats, sharing merely 144 ASVs with oil and 1 ASV with cores. Taken together, our microbial modulation strategy could selectively biostimulate a wide range of bacterial species from sandstone cores and oil phase, which emerged and were enriched in PW (8, 9, 10).

LEFSe and STAMP analyses identified the presence of significantly different taxa among various stages of PW (Fig. 2b, c, Supplementary Fig. S6). Genera enriched in PW (5, 6, 7) primarily belonged to *Proteobacteria* and *Firmicutes*, including *Pseudomonas*, *Tissierella*, *Lachnoclostridium*, *Alcaligenes*, and *Fusobacterium*. Many taxa affiliated with *Desulfobacterota* and *Synergistota* showed significantly higher relative abundance in August (LDA score > 3; Fig. 2b). At the latter stage of PW (9, 10), *Campilobacterota*, *Deferribacterota*, *Desulfobulbales*, and *Verrucomicrobiota* were evidently more abundant. *Desulfovibrio*, *Sulfurospirillum*, and *Shewanella*, as the main biomarkers at the genus level for differentiating the PW of August, September, and October, were significantly enriched in PW (8) and PW (9, 10), respectively (FDR adjusted *p-*value < 0.05, Welch’s *t*-test; Fig. 2c).

### Bacterial co-occurrence network analysis

**Fig. 3:**
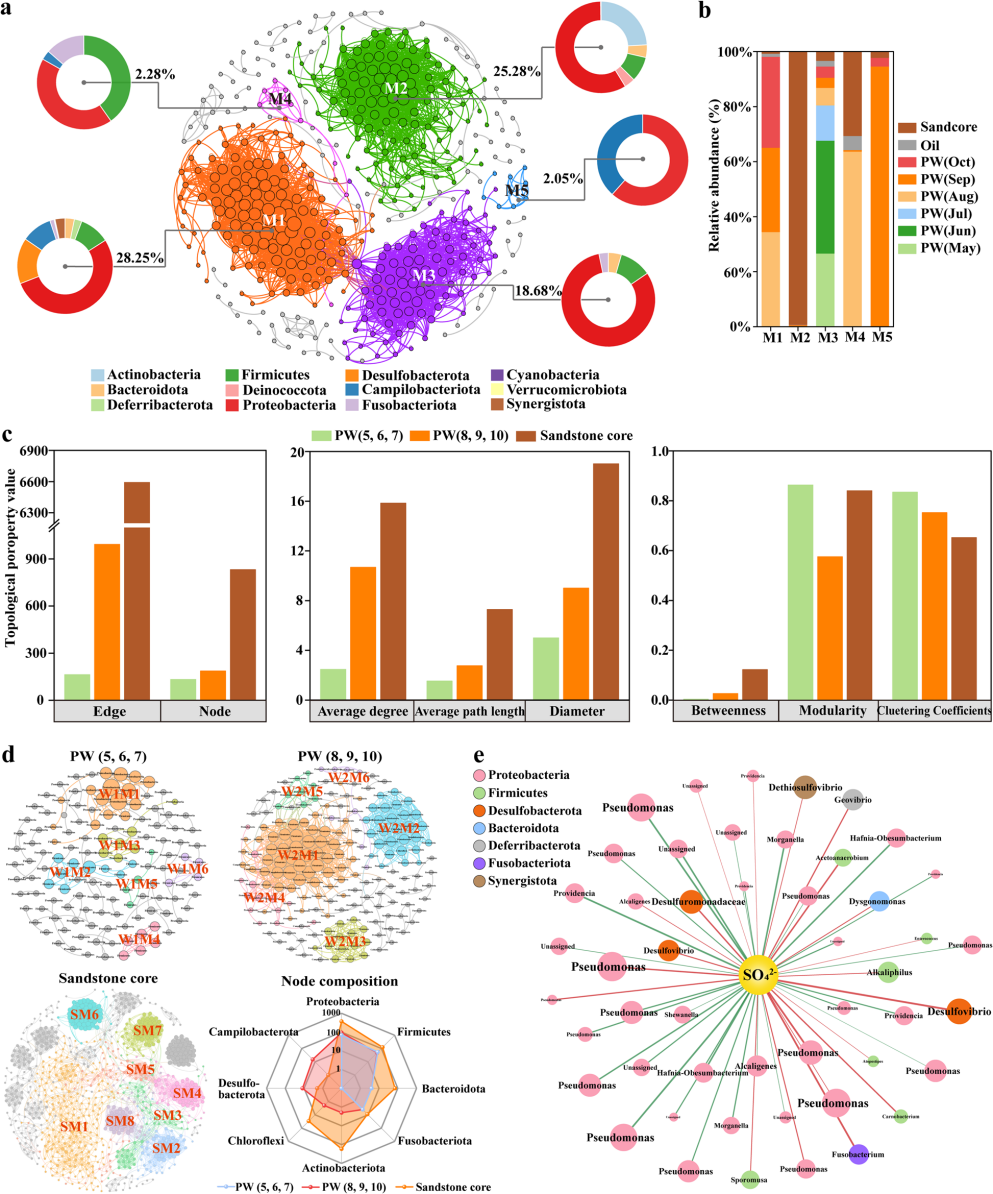
Network analysis revealing microbial co-occurrence patterns and ecological characteristics of different subsurface habitats based on the top ASVs (relative abundance > 0.01%).** a** The co-occurrence network and module division of the five main modules (M1–M5) when considering all samples. Distinct modules represent differences in niches. Taxonomic compositions of modules were estimated based on the composition of ASV abundance-weighted nodes within the module. **b** The distribution of distinct groups of environmental samples in each module reveals which niches (modules) the samples from various habitats primarily represented. **c** Topological properties of PW (5, 6, 7), PW (8, 9, 10), and sandstone core subnetworks. **d** Co-occurrence patterns of the subnetworks in specific habitats and their node compositions. **e** Correlation network showing the significant link between microbial species and electron acceptors (sulfate and nitrate). A connection represents a strong and significant correlation with Spearman’s coefficient of |*r*| > 0.7 and *p-*value < 0.01. The genera of the ASVs are listed in nodes, and the colors of nodes signify the phyla with which the ASVs are affiliated. Red and green edges denote positive and negative correlations, respectively. Node size and linewidth correspond to the relative abundance of ASVs and the absolute value of correlation coefficients, respectively

The bacterial co-occurrence network covering all types of samples was constructed, which consisted of 439 nodes and 5093 edges after removing unconnected vertices (Fig. 3a). The modularity value of the network was 0.643, much higher than 0.4, indicating that the network structure is highly modular [73]. Over 75% of nodes were attributed to the 5 domain modules (M1–M5) out of the total 43 modules (Fig. 3a). ASVs within a module interact and communicate intimately, while they are less connected with those outside the module [74]. The five modules displayed completely variable bacterial community compositions linked with specific habitats (Fig. 3a). Module 1 comprised substantial portions of *Desulfobacterota* (15.5%) and *Campilobacterota* (10.3%), which were the typical bacterial representatives of PW during the succession period. Module 2 resembled the community composition pattern of lithospheric minerals due to its high species diversity and the dominance of *Actinobacteria* (24.1%). Module 3 was populated by a considerably higher relative abundance of *Proteobacteria* (82.5%) with a simple community structure, similar to the composition of initial PW. Our speculations were confirmed by examining habitat distributions in various modules (Fig. 3b), which revealed that the ASV features from PW (5, 6, 7), PW (8, 9, 10), and minerals were essentially clustered into discrete modules. M1 principally represented PW (8, 9, 10), M2 was the typical niche of lithospheric minerals, and M3 was specific to PW (5, 6, 7). The evident modular distribution is indicative of high niche differentiation among the habitats of PW (5, 6, 7), PW (8, 9, 10), and lithospheric minerals, providing evidence that our modulation induced the evolution of bacterial community by modifying environments and constructing niches, known as “niche-construction theory” [75]. Overlapped habitats between the PW of August and sandstone cores were observed in M4, whereas M5 specifically referred to the PW of September.

Considering the topological properties of each habitat’s subnetwork (Fig. 3c, d, Supplementary Fig. S7), immense dissimilarities in ecological characteristics and bacterial interaction patterns were discovered. All subnetworks exhibited the features of complex systems and nonrandom construction by showing high modularity (0.575–0.840) and clearly different topological indices (e.g., degree distribution) from their corresponding random networks (Supplementary Fig. S8). The bacterial co-occurrence network of lithospheric minerals was much larger and more connected than that of PW, with more edges and nodes, a higher average degree, and a larger diameter. The network of PW (5, 6, 7) had exceedingly small average path length but was highly clustered, exhibiting the typical features of “small world” [76]. This was corroborated by our calculation of “small-world coefficients” (Supplementary Table S4), indicating that the delivery and exchange of resources are highly efficient and fast-reaching throughout the network [74, 77]. Accordingly, we conjecture that the primitive lithospheric biosphere was susceptible to disturbance, implying that the invasion of exogenous bacteria and environmental changes would potentially destroy the entire microbial network and community structure. Compared with the subnetwork of lithospheric minerals, higher clustering coefficients, shorter average path length, and lower betweenness were observed in that of PW, which reflected that the microbial interaction in PW was more intricate and stronger [78]. Additionally, subnetworks revealed environment-specific co-occurrence relationships. The interspecies coexistence of PW (5, 6, 7) was within or between the phyla *Proteobacteria* and *Firmicutes*, whereas *Campilobacterota* and *Desulfobacterota* enriched in PW (8, 9, 10) primarily coexisted with *Proteobacteria*, *Firmicutes*, and *Actinobacteria* (Supplementary Fig. S7). Compared to PW, lithospheric minerals showed distinct co-occurrence relationships, deriving from diverse taxonomic members of *Actinobacteria*, *Bacteroidota*, *Chloroflexi*, and *Acidobacteriota*.

The correlation network analysis of microbe–environmental factors showed that numerous ASVs were strongly correlated with ${\mathrm{SO}}_{4}^{2-}$(Spearman’s correlation coefficient: *|r|* > 0.7 and *p*-value < 0.01; Fig. 3e). The majority of SRB were significantly and positively correlated with ${\mathrm{SO}}_{4}^{2-}$, including *Desulfuromonadaceae*, *Desulfovibrio*, and *Dethiosulfovibrio* (*r* = 0.81, 0.81, and 0.74, respectively). The genus *Geovibrio*, which was reported to be capable of using sulfur or Fe (III) as electron acceptors [79], also exhibited a highly positive correlation with ${\mathrm{SO}}_{4}^{2-}$ (*r* = 0.79). Most of the negatively correlated ASVs with ${\mathrm{SO}}_{4}^{2-}$ were affiliated with *Proteobacteria*, such as *Providencia* and *Shewanella*. Positive and negative correlations with ${\mathrm{SO}}_{4}^{2-}$ were both involved in the genus *Pseudomonas*, reflecting that members of *Pseudomonas* could instead undertake versatile metabolic functions. Additionally, the deficit in significant correlations between ASVs and nitrate under the rigorous significance test suggested that sulfate appeared to play a relatively more crucial role in shaping bacterial community structure than nitrate.

### The release of N and S governing bacterial community succession

**Fig. 4:**
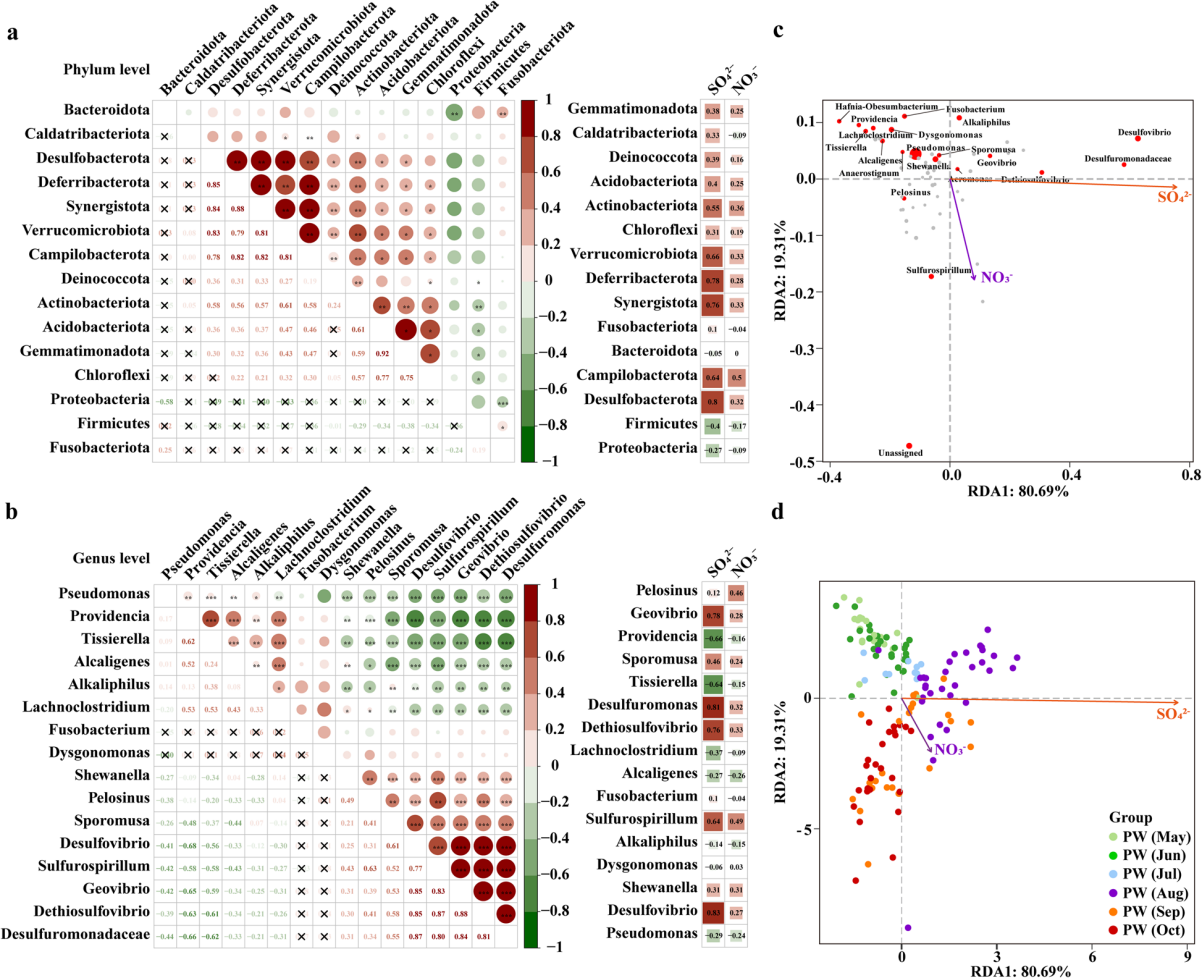
Change patterns of key taxonomic abundance across the succession driven by the advent of electron acceptors.** a** Abundance correlations within the dominant taxa at the phylum level and correlations between taxa abundance and electron acceptors. **b** Correlation analysis at the genus level. The size and color gradients of circles denote Spearman’s correlation coefficient. Statistical significance of *P*-values was expressed as ***P < 0.001, **P < 0.01, *P < 0.05. **c** The transformed redundancy analysis (tRDA) showing the relevance between genera and electron acceptors. **d** tRDA showing the electron acceptor-driven shift of microbial community structure of PW samples

Correlations within the top 15 most abundant taxa and correlations between taxa and environmental factors were calculated to examine the abundance relevance among core species in response to varying sulfate and nitrate concentrations (Fig. 4). The advent of electron acceptors enabled members of a functional group (groups of taxa potentially capable of performing a particular metabolic function) to show a similar response, suggesting microbial adaptation to the environmental and niche changes [64]. For instance, sulfur respiration-related genera, including *Geovibrio*, *Desulfovibrio*, *Sulfurospirillum*, *Dethiosulfovibrio*, and *Desulfuromonadaceae*, were positively correlated with each other (Spearman’s *r* = 0.77 to 0.88, *P* < 0.001). The correlation and redundancy analyses showed that nitrogen- and sulfur-cycling genera became more abundant with the increasing availability of sulfate and nitrate, whereas the proportions of *Pseudomonas* and *Tissierella*, the dominant taxa in initial PW (5, 6, 7), were diminished as electron acceptors became available (Fig. 4b, c). Some abundant taxa were found to be insensitive to electron acceptor changes, such as *Fusobacterium*, *Dysgonomonas*, and *Alkaliphilus* ($|r|$ < 0.2). Additionally, following the intervention, divergent succession trajectories were observed for PW bacterial populations, which evolved toward the increase in sulfate during August and then transitioned to the direction of increasing nitrate during September and October (Fig. 4d). This proved that sulfate governed bacterial community succession in August, but nitrate served as the controlling factor affecting community structure in September and October.

### Functional profiles for the microbiome of production water

**Fig. 5:**
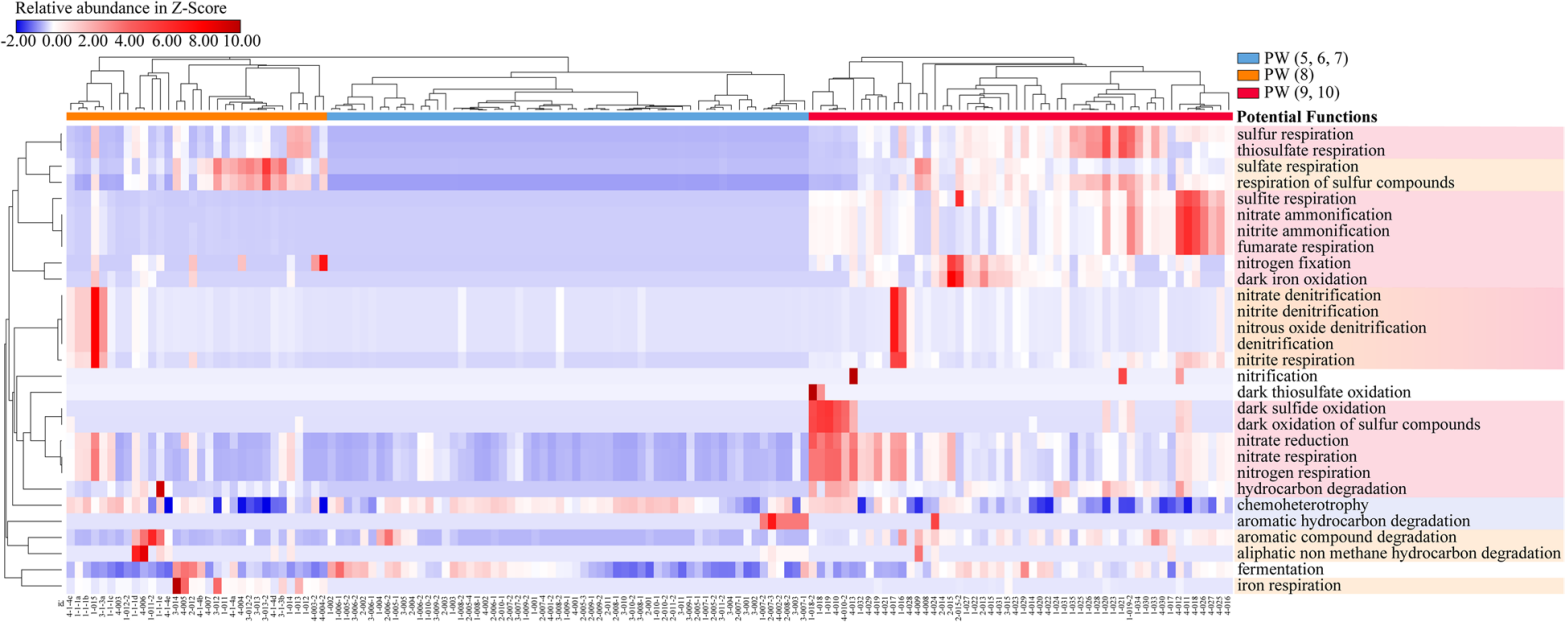
Community functional profiles predicted based on the FAPROTAX database. Darker red colors correspond to higher relative abundances of functional bacterial groups possessing the function. The prominent functions at each stage of the PW community were consolidated and shadowed using the corresponding color

Metabolic functions of oil field microbial populations at various stages of PW were preliminarily predicted by alignment of the taxa-based functional database FAPROTAX (Fig. 5). The bacterial community of initial PW (5, 6, 7) primarily performed the metabolisms of chemoheterotrophy and aromatic hydrocarbon degradation, whereas they were endowed with more functions related to nutrient cycles (sulfur, nitrogen, and iron respiration) after exogenous *Pseudomonas* injection. Specifically, the bacterial community of PW (8) tended to participate in sulfate respiration/reduction, sulfur respiration, and aromatic compound degradation. Afterward, nitrate reduction (e.g., nitrate ammonification, denitrification) and sulfur/sulfide oxidation became significantly more prevalent and active in September and October (FDR adjusted *p-*value < 0.05, Welch’s *t*-test; Supplementary Fig. S9).

**Fig. 6:**
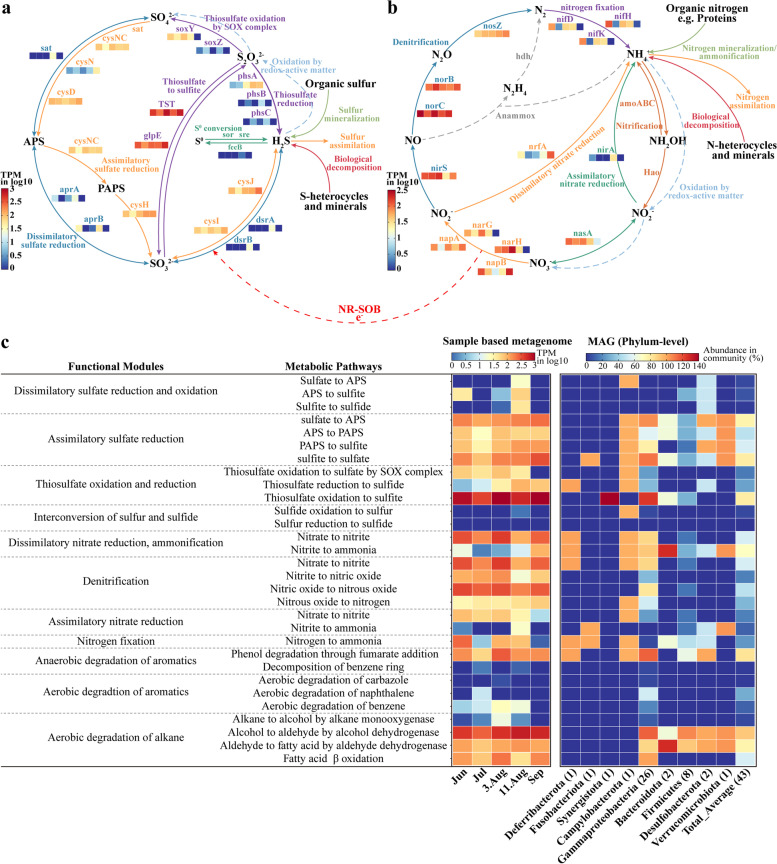
Metabolic potentials of PW microbiomes in sulfur and nitrogen cycles as well as hydrocarbon biodegradation. The relative abundance of signature genes encoding enzymes associated with the respective pathway in PW microbiomes from various stages was expressed as TPM (total transcripts per million) and represented by color blocks that correspond to the PW of June, July, August 3rd, August 11th, and September (from left to right). **a** Metabolic potential predictions of sulfur-cycling pathways, including dissimilatory sulfate reduction, thiosulfate oxidation and reduction, assimilatory sulfate reduction, and sulfide oxidation. **b** Metabolic potential predictions of six distinct nitrogen-cycling pathways, including dissimilatory nitrate reduction, denitrification, assimilatory nitrate reduction, nitrogen fixation, nitrification, and anaerobic ammonia oxidation (anammox). The genes responsible for nitrification and anammox were not detected. Multiple NR-SOB can channel electron transfer between nitrate reduction and sulfide oxidation. **c** The metabolic capacity of MAG members and PW microbiomes from various stages. The left heatmap shows the aggregated abundance of all signature genes encoding the individual metabolic pathway in PW microbiomes. The right heatmap gives the presence percentage of signature genes involved in metabolic pathways across 43 reconstructed high-quality draft genomes spanning 9 phyla. Abundance was normalized by the completeness of MAGs to eliminate the influence of incomplete assembly on the prediction of gene presence

To further unravel the concrete process of modulating the lithospheric microbiome, we applied metagenomic analysis to profile the abundance of genes involved in sulfur and nitrogen cycling, alkane and aromatic degradation based on both assembled short contigs and MAGs (Fig. 6). In accordance with the FAPROTAX prediction results, genes involved in metabolic pathways of sulfur cycling in the microbiome of August were widely detected and exhibited higher abundance than those in other phases (Fig. 6a). Especially, the microbiome of August contained the complete repertoire of genes for dissimilatory sulfate reduction pathway (*sat*, sulfate adenylytransferase; *aprAB*, adenylylsulfate reductase; *dsrAB*, dissimilatory sulfite reductase), which underpinned the utilization of sulfate as electron acceptors. However, these genes were absent or at much lower abundance elsewhere. These findings were further supported by the transcriptional level of gene expression (Supplementary Fig. S10). The relative expression levels of *dsrAB* in the last step of dissimilatory sulfate reduction were significantly upregulated after biostimulation (*P* < 0.05, one-way ANOVA with Fisher’s LSD post hoc test). In addition, genes responsible for assimilatory sulfate reduction were consistently detected throughout all PW samples, and the relative abundance of sulfite reductase-encoding genes (*cysIJ*) required in the last step was increased by 0.8~2.8 folds in the microbiota of September. Furthermore, thiosulfate reduction was prone to occur in the PW after the intervention owing to the higher abundance of *phsA* (thiosulfate reductase) in PW (8, 9), which was in excess of five times that in PW (5, 6, 7). Consequently, it was obvious that the abundance of genes associated with sulfur metabolism was enhanced after microbial intervention in comparison to original petroleum reservoir microbiomes. The microbiome of petroleum reservoirs in August possessed the most diverse sulfur-cycling metabolism characterized by dissimilatory sulfate reduction, whereas with the depletion of sulfate, the drivers of sulfur cycling primarily depended on assimilatory sulfate reduction and thiosulfate reduction.

Nitrogen-cycling genes, specifically those involved in denitrification and dissimilatory nitrate reduction to ammonium (DNRA), were ubiquitous in PW samples (Fig. 6b). Genes responsible for denitrification were the most abundant among all nitrogen-cycling pathways, revealing that denitrification is a core and conserved nitrogen metabolic pathway for lithospheric microorganisms. By contrast, the absence of ammonia monooxygenase-encoding genes (*amoABC*) and anammox genes (*hdh*) indicated that nitrification and anammox were rarely carried out in deep lithospheric environments, consistent with the previous report from anoxic aquifers [14]. Notably, a considerably higher relative abundance of *nrfA* (5- to 49-fold increase) encoding cytochrome c-552 in the final step of DNRA (nitrite reduction to ammonia) was identified in September’s microbiome, suggesting that DNRA was presumably more prevalent and the dominant pathway for nitrate/nitrite reduction in September. RT-qPCR results also confirmed the significantly increased transcript abundance of key genes for denitrification and DNRA with *napA* and *nosZ* upgrading by 22 and 27 folds, respectively, after long-term intervention, compared to the initial reservoir stage (Supplementary Fig. S10). Lower potential for nitrogen fixation was found in the PW after the intervention due to the rare detection of Nitrogenase (*nifDKH*), but considerably more nitrogen-fixing microorganisms were expected to inhabit the initial reservoir. With regard to the metabolic potentials of hydrocarbon degradation, alkane- and aromatic-degrading genes were the most abundant in the microbiome of August (Fig. 6c), which could result from the prevalent occurrence of SRB members.

To link metabolic functions to microbes, we retrieved 43 non-redundant near-complete MAGs comprising the majority of bacterial phyla (Supplementary Fig. S12). The metabolic capacity of MAGs was determined by assessing the presence of key genes (Fig. 6c, Supplementary Dataset S2). Numerous MAGs had the collective capacity for undertaking the aforementioned metabolic pathways such as assimilatory sulfate reduction (24/43, 24 of the 43 genomic bins), DNRA (23/43), and alkane degradation (22/43) (Supplementary Dataset S2). Marker genes (*sat*, *aprAB*, and *dsrAB*) encoding complete dissimilatory sulfate reduction pathway enzymes were pervasive in *Desulfobacterota* MAGs (50%). *Campylobacterota* MAGs harbored a rich repertoire of genes encoding key enzymes in nitrogen and sulfur metabolic pathways, particularly DNRA (100%). The metabolic capacity of *Gammaproteobacteria* members was extremely versatile, which putatively involved hydrocarbon degradation and engaged in nitrogen and sulfur cycling.

### Prominent effects on oil properties and oil recovery

Field trials validated the promising prospects of our exogenous bacterial modulation for heavy oil viscosity reduction and recovery enhancement. The cumulative oil production for wells #1 and #4 was substantially enhanced after the microbial modulation (Fig. 1b), increasing by 610 and 470 tons (approximately 4484 and 3455 barrels), respectively, till October. SARA analysis of heavy oil showed that the average content of asphaltenes (S,N-heterocycles) significantly decreased from 15.31 to 3.88% (*t*-test, *P* < 0.05; Supplementary Fig. S14 and Table S2), and the content of sulfur and metal elements also dropped by orders of magnitude (Supplementary Table S3). This result indicated recalcitrant heterocyclic hydrocarbons were largely degraded, which was the major contribution to the improved oil recovery.

## Discussion

Exploration of associations between element cycling and microbial metabolic activities provides a unique portal into the operation of microbial life in deep crustal environments. This study sheds light on the succession law of microbial communities in heavy oil reservoirs following our modulation and highlights the critical role of the release and transformation of N/S elements in affecting lithospheric biogeochemical cycling. A field microbial regulatory practice for enhanced heavy oil recovery was undertaken, revealing the mechanism of the manipulation of exogenous heterocycle-degrading bacteria to selectively stimulate indigenous functional groups related to sulfur and nitrogen metabolism. We propose naming this sort of bacteria with exceptional capabilities of dissociating heteroatoms from heterocycles or minerals the “bioredox triggers”.

**Fig. 7:**
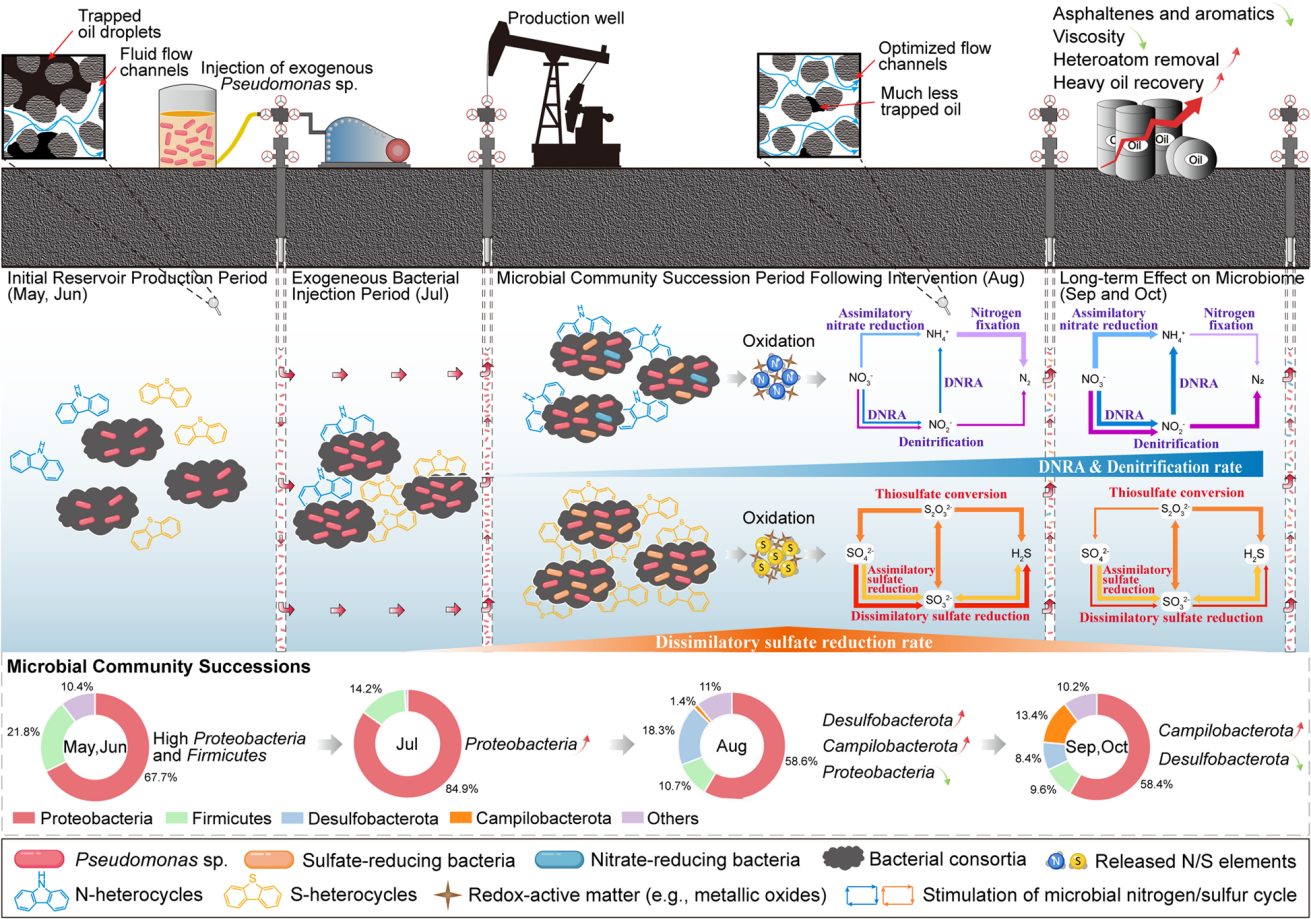
Schematic diagram of exogenous bacteria (“bioredox trigger”) modulating lithospheric indigenous microbial communities and element cycling. The early primary reservoirs (May, June) were primarily dominated by *Proteobacteria* and *Firmicutes* where electron acceptors were deficient. Our strategy of exogenous single-species manipulation was implemented in July by injecting heterocycle-degrader *Pseudomonas* strains into the subsurface. The N/S elements were in situ released and transformed to replenish extremely scarce electron acceptors in the lithosphere, activating the lithospheric N/S cycling and inducing microbial community succession (after Aug)

In detail, pristine subsurface environments are nutrient deficient, and thus microbial-driven heterocycle degradation and element cycles are stagnant. Upon the injection of exogenous *Pseudomonas* sp., N/S elements in heterocycles were dissociated and liberated as reduced inorganic nitrogen and sulfur compounds during bioaugmented heterocycle decomposition (Fig. 7). N/S chemicals might be oxidized by redox-active matter (e.g., ferrihydrite, manganese oxides, perchlorate) or microbe-mediated (biotic and abiotic) reactions to nitrate and sulfate (or their intermediate substances), when transported by pore water to zones with high ORP (oxidation/reduction potential) in highly heterogeneous oil reservoir environments (Supplementary Results and Fig. S15). These oxidative substances will serve as electron acceptors, thereby establishing multiplex metabolic niches in petroleum reservoirs. Changes in reservoir niches and the appearance of inorganic electron acceptors resulted in a rapid proliferation of SRB and NRB, which in turn accelerated the biodegradation of heterocycles [80–82]. Diversity analysis showed that the taxonomic diversity of microorganisms in PW was significantly enhanced. This phenomenon is possibly the consequence of the promoted functional redundancy, which means that the advent of new metabolic niches led to rising microbial clades with similar functions competing and cooperating within the niches, better buffering against taxonomic shifts caused by the intervention [64, 83]. Furthermore, the phylogenetic and functional diversity of the lithospheric mineral microbiome was significantly higher than that of the PW microbiome. The view was supported by our delineation of co-occurrence subnetworks, which showed that more modules (dissimilar niches) were categorized in the network of lithospheric minerals (Supplementary Fig. S7).

We also discerned minor transitions in bacterial community compositions and functions of PW following the intervention. SRB and genes responsible for dissimilatory sulfate reduction were more prevalent in the former phase (Aug), whereas SRB subsequently diminished, and the reservoir was prone to the dominance of anaerobic respiration of nitrate and nitrite by NRB or NR-SOB (in Sep and Oct). The trend of SRB was in good concordance with the change in H_2_S concentration in reservoirs (Supplementary Fig. S2a), which increased dramatically during August and then declined after mid-September. Possible reasons for this microbial succession pattern could include the following: (1) Nitrogen content is generally much lower than sulfur content in petroleum asphaltenes and thus less nitrogen was released [84]; (2) Metagenomics and previous studies indicated that SRB, primarily affiliated with *Desulfobacterota*, possessed larger potentials for S-heterocycle biodegradation such as benzothiophene [80, 81], whereby the release of sulfur was preferentially promoted; (3) The redox potentials (${\mathrm{E}}^{0\mathrm{^{\prime}}}$) of ${\mathrm{NO}}_{3}^{-}$/${\mathrm{NO}}_{2}^{-}$ (+430 mV) and ${\mathrm{NO}}_{2}^{-}$/${\mathrm{NH}}_{4}^{+}$ (+440 mV) are much higher than that of ${\mathrm{SO}}_{4}^{2-}$/${\mathrm{HSO}}_{3}^{-}$ (−516 mV) and ${\mathrm{HSO}}_{3}^{-}$/${\mathrm{HS}}^{-}$ (−110 mV), and therefore nitrate reduction and reduced sulfur oxidation are thermodynamically favored [21], resulting in increasing sulfate accumulation but little residual nitrate at inception; (4) The rapid proliferation of SRB caused fierce trophic competition among species, the depletion of sulfate, and elevated concentrations of hydrogen sulfide, inhibiting the growth of SRB; (5) SRB and dissimilatory sulfite reductase can be competitively inhibited with the accumulation of nitrite (or nitrate) in PW (9, 10).

Co-occurrence network analysis revealed that the release of N/S markedly affected bacterial community structure and created new metabolic niches. Co-occurrence of ASVs in a module is indicative of similar niche adaptation and interspecies links signify their competitive or cooperative relationships [85]. Bacterial co-occurrence patterns indicated that the emergent bacteria, primarily affiliated with *Desulfobacterota* and *Campilobacterota*, could co-exist with *Gammaproteobacteria* (e.g., *Pseudomonas*) and *Firmicutes*, which suggested that the directed stimulation of functional groups could rely on the synergistic modulation of electron acceptor provisioning and interspecies symbiosis (such as mutualism, commensalism, or competition). The analysis of topological properties indicated that the magnitude and connectedness of the subnetwork of the PW microbiota (8, 9, 10) showed an obvious increase after the intervention. The increase in connections could partially stem from community successions and the increased species diversity. Alternatively, the appearance of available nutrients and electron acceptors (e.g., ${\mathrm{NO}}_{3}^{-}$, ${\mathrm{SO}}_{4}^{2-}$) induced more convoluted and stronger interspecies cooperation or competence for nutrient acquisition, which is the canonical trait in N/S-transforming microbial systems. For example, nitrifiers *Nitrosopumilus* spp. facilitate the growth of anammox bacteria (*Ca Scalindua* spp.) by generating nitrite from ammonia oxidation, but also compete for ammonia alongside them [1]. It is worth noting that higher community stability was speculated to be established for PW (8, 9, 10) due to the weaker interactions (smaller clustering coefficient) and higher connectedness, implying that the introduction of exogenous bacteria enabled lithospheric environments to remain comparably more resistant and resilient to environmental perturbations. Field experiments validated the extraordinary effectiveness of our microbial modulation in viscosity reduction, heteroatom removal, and incremental yield of heavy oil.

## Conclusions

This study disentangled the microbial community succession dynamics and revealed that the active biogeochemical sulfur and nitrogen cycles induced by exogenously introduced heterocycle-degrading bacteria could significantly shift the structure and function of the microbiome and shape new metabolic niches in deep subterranean environments. This approach breaches the obstacles of conventional direct nutrient injections, such as the rapid depletion of electron acceptors during their transportation and failure to achieve targeted activation of functional microorganisms. Meanwhile, the proposal of this engineered microbial intervention strategy and the revelation of the modulation mechanism will inform future studies on interconnections between microbial activities and lithospheric biogeochemical cycling. Our findings also offer prospects for expanding biotechnological applications for recovering the vast quantity of heavy oil resources that would otherwise remain unexploited. Further efforts are needed to comprehend the microbial community dynamics and their relationships with biogeochemistry in the lithosphere more deeply and to unravel mechanisms of interspecies interactions.

## Supplementary Information

**Additional file 1:** **SUPPLEMENTARY METHODS** include Exogenous bacterial inoculum, Sandstone core sampling and processing, production water sampling and processing; DNA extraction and sequencing; Amplicon generation and sequencing; and Enumeration of bacterial diversity in sandstone cores and PW. **SUPPLEMENTARY RESULTS** include Cell densities in petroleum reservoir environments; Hidden anoxic sulfur and nitrogen cycles for electron acceptor generation; Assessment and analysis of the biostimulation performance on oil recovery; and Assessment on effects of reagent and laboratory contamination. **Figure S1.** Schematic diagram of sandstone core sampling and processing for microbiome analysis. **Figure S2.** The temporal variations in major chemical forms of sulfur and nitrogen during the entire period of field trials. **Figure S3.** The temporal variations of bacterial compositions across all PW samples. **Figure S4.** The bacterial compositions of sandstone cores across different wells. **Figure S5.** The archaeal compositions of partial PW and sandstone core samples. **Figure S6.** Significantly different phyla among various stages of PW. **Figure S7.** Co-occurrence subnetworks in various habitats and the node composition. **Figure S8. **The discrepancies of degree distributions between subnetworks and their corresponding random networks. **Figure S9.** Significantly different functions among various stages of PW’s microbiomes predicted by aligning to the FAPROTAX database. **Figure S10.** Fold-change of key genes’ relative expression by RT-qPCR. **Figure S11.** Summary of recovered MAGs. **Figure S12.** Taxonomy and phylogenetic tree of high-quality MAGs. **Figure S13.** Tracking of the variations of key functional strains and bacterial cell density in PW along the microbial modulation. **Figure S14.** SARA analysis of heavy oil component changes from field trails by our exogenous bacterial modulation. **Figure S15.** Hidden sulfur oxidation processes for oxidation of reduced sulfur compounds to sulfate. **Figure S16.** Summary of key genes related to sulfur, nitrogen cycling and hydrocarbon degradation that were investigated in the metagenomics analysis. **Figure S17.** The process flow diagram (PFD) for the preparation of exogenous Pseudomonas strain inoculum. **Figure S18**. Assessment of potential reagent and laboratory contaminations and effects on biological samples. **Figure S19.** Venn diagram and taxonomic compositions at different taxonomic classification levels. **Table S1.** Primers sequences and RT-qPCR programs used in this study. **Table S2.** SARA and viscosity analysis of heavy oil from field trails before and after exogenous bacterial intervention. **Table S3.** Element analysis of heavy oil from field trails before and after the exogenous bacteria intervention. **Table S4.** The degree of small-world properties of subnetworks. **Table S5.** The physical characteristics and bacterial cell densities of various petroleum reservoir habitats.

**Additional file 2:** **Dataset S1.** Geochemical measures and bacterial abundance of PW, oil and sandstone cores.

**Additional file 3:** **Dataset S2.** Summary of the taxonomy and gene presence of metagenome-assembled genomes and gene relative expression abundance by RT-PCR.

## Data Availability

All data of the raw amplicon sequencing and metagenomic sequencing as well as the assembled genomes reported in this study are deposited in the National Center for Biotechnology Information (NCBI) Sequence Read Archive under BioProject accession no. PRJNA838702. The R scripts for correlation and network analyses are publicly available on GitHub at https://github.com/shunyao-nku/RedoxTrigger.

## Abbreviations

IMEOR: Indigenous microbial enhanced oil recovery
PW: Production water
SARA: An acronym for saturates, aromatics, resins, and asphaltenes
CFU: Colony-forming unit
ASV: Amplicon sequence variants
OTU: Operational taxonomic units
ANI: Average nucleotide identity
MAGs: Metagenome-assembled genomes
PCoA: Principle coordinate analysis
tb-RDA: Transformed-based redundancy analysis
LEFSe: Linear discriminant analysis effect size
SRB: Sulfate- or/and sulfite-reducing bacteria
NRB: Nitrate- or/and nitrite-reducing bacteria
NR-SOB: Nitrate-reducing–sulfide oxidizing bacteria
DNRA: Dissimilatory nitrate reduction to ammonium
RT-qPCR: Reverse transcription real-time quantitative PCR
dsr: Dissimilatory sulfite reductase
napA: Periplasmic nitrate reductase
nosZ: Nitrous oxide reductase
sat: Sulfate adenylyltransferase
apr: Adenylylsulfate reductase
TPM: Transcripts per million
