# Supplementary material for: N/S element transformation modulating lithospheric microbial communities by single-species manipulation
Source: Microbiome. 2023 May 16;11:107. doi: 10.1186/s40168-023-01553-7 (PMC10186816; doi:10.1186/s40168-023-01553-7)
Supplement: Supplementary file 2 — Additional file 1: SUPPLEMENTARY METHODS include Exogenous bacterial inoculum, Sandstone core sampling and processing, production water sampling and processing; DNA extraction and sequencing; Amplicon generation and sequencing; and Enumeration of bacterial diversity in sandstone cores and PW. SUPPLEMENTARY RESULTS include Cell densities in petroleum reservoir environments; Hidden anoxic sulfur and nitrogen cycles for electron acceptor generation; Assessment and analysis of the biostimulation performance on oil recovery; and Assessment on effects of reagent and laboratory contamination. Figure S1. Schematic diagram of sandstone core sampling and processing for microbiome analysis. Figure S2. The temporal variations in major chemical forms of sulfur and nitrogen during the entire period of field trials. Figure S3. The temporal variations of bacterial compositions across all PW samples. Figure S4. The bacterial compositions of sandstone cores across different wells. Figure S5. The archaeal compositions of partial PW and sandstone core samples. Figure S6. Significantly different phyla among various stages of PW. Figure S7. Co-occurrence subnetworks in various habitats and the node composition. Figure S8. The discrepancies of degree distributions between subnetworks and their corresponding random networks. Figure S9. Significantly different functions among various stages of PW’s microbiomes predicted by aligning to the FAPROTAX database. Figure S10. Fold-change of key genes’ relative expression by RT-qPCR. Figure S11. Summary of recovered MAGs. Figure S12. Taxonomy and phylogenetic tree of high-quality MAGs. Figure S13. Tracking of the variations of key functional strains and bacterial cell density in PW along the microbial modulation. Figure S14. SARA analysis of heavy oil component changes from field trails by our exogenous bacterial modulation. Figure S15. Hidden sulfur oxidation processes for oxidation of reduced sulfur compounds to sulfate. Figure S16. Summary of ke [file 40168_2023_1553_MOESM1_ESM.pdf]

Supplementary Information for

N/S Element Transformation Modulating Lithospheric Microbial Communities by Single-species Manipulation

Shun Yao[a,1], Tianzhi Jin[a,1], Lu Zhang[a], Yong Zhang[a], Rui Chen[b], Qian Wang[b], Mingjie Lv[b], Chuxiao Hu[a], Ting Ma[a,*], Wenjie Xia[a,1,*]

[a] Key Laboratory of Molecular Microbiology and Technology, Ministry of Education, College of Life Sciences, Nankai University, Tianjin 300071, P.R. China;

[b] Institute of Crop Germplasm and Biotechnology, Tianjin Academy of Agricultural Sciences, Tianjin 300381, China

* Corresponding author. E-mail: Wenjie. Xia (wenjie.xia@nankai.edu.cn); Ting Ma, (tingma@nankai.edu.cn)

This PDF file includes the following items:

SUPPLEMENTARY METHODS

SUPPLEMENTARY RESULTS

SUPPLEMENTARY FIGURES (S1-S19)

SUPPLEMENTARY TABLES (S1−S5)

REFERENCES

Other Supplementary Materials for this manuscript include the following:

Dataset S1. Geochemical measures and bacterial abundance of PW, oil and sandstone cores.xlsx

Dataset S2. Summary of the taxonomy and gene presence of metagenome-assembled genomes and gene relative expression abundance by RT-PCR.xlsx

Contents

Supplementary Methods 1

Exogenous bacterial inoculum 1

Sandstone core sampling and processing 1

Production water sampling and processing 2

DNA extraction and sequencing 2

Extraction of genome DNA from production water 2

Extraction of genome DNA from sandstone cores 3

Amplicon generation and sequencing 3

Enumeration of bacterial density in sandstone cores and PW 4

Enumeration of culturable aerobes and anaerobes by plate counts 4

Enumeration of bacteria by quantitative PCR 4

Supplementary Results 6

Cell densities in petroleum reservoir environments 6

Hidden anoxic sulfur and nitrogen cycles for electron acceptor generation 6

Assessment and analysis of the biostimulation performance on oil recovery 8

Assessment on effects of reagent and laboratory contamination 9

Supplementary Figures 11

Supplementary Tables 30

References 32

Supplementary Methods

Exogenous bacterial inoculum

Pseudomonas sp. WJ6 (GenBank No. KF155141) was deposited in the China Central Microbiological Culture Collection Center with the deposit number of CGMCC 4402, which is one of the 86 strains isolated from heavy oil-contaminated soils. The morphological, physiological, and phylogenetic characteristics of this isolate were well elaborated previously [1]. *Pseudomonas*, as a well-recognized Gram-negative S,N-heterocycle degrader, has demonstrated great potential for degrading asphaltenes and heavy oil [2–4]. Furthermore, the carbazole-degrading gene cluster (*carAaBbCAcAdDFE*) and plasmid (pCAR1), as well as biodesulfurization and biodenitrogenization pathways, were successively discovered in *Pseudomonas* lineages [5–7].

After a 36-hour large-scale culture in sterile LB medium at 35°C, dense bacterial cells were harvested by pumping the bacterial fermentation liquid (~10^9 CFU mL^{-1}) through filtration bags and washing with 0.01M sterile PBS, as illustrated in Fig. S17. The dry cells were then delivered on-site and mixed with local clean groundwater in two tandem blending tanks to prepare cell suspensions as inoculum for biostimulation trails. The cell density of the injection inoculum was diluted to roughly 2×10^7 CFU mL^{-1} at a 50-fold dilution of the original fermentation liquid. Sulfate and nitrate concentrations in utilized groundwater were determined to be negligible at 0.011 and 0.000 g/L, respectively. The inoculum was prepared and infused four times through the water injection well into the oil-bearing strata (50 m^3 on June 19th, 50 m^3 on June 20th, 58 m^3 on July 11th, and 10 m^3 on July 26th).

Sandstone core sampling and processing

The depth of sandstone core samples ranged from 185 to 205.5 meters below the ground surface. After the coring location was determined by geological staff, drilling operations were commenced, accompanied by the use of drilling fluids, until rock bit approaches the designed oil-bearing layer (at a depth of 180 m) according to the previous local geological survey. The drill bit was then replaced by a coring barrel to collect the first core segment for lithological analysis, ensuring the positional accuracy of coring. Once coring sites and depths were confirmed, consecutive sealed coring was performed to collect enough core segments until approaching bedrocks (the base of the oil leg). The length of coring segments had to be less than 9 m, which is the maximum of coring barrels; meanwhile, core truncation is preferably designed to occur in mudstone sections instead of sandstone to facilitate cutting and lithological analysis. Core segments encased in coring barrels were swiftly scraped with a sterile chisel blade to remove superficial drilling fluids and fragments. Staff geologists precisely measured and logged the length and depths of core segments that were

placed in sequence as they were retrieved from the subsurface.

Fresh core segments underwent a series of on-site processing before being transported to the laboratory. Firstly, core segments were aseptically split longitudinally into two sections using specialized cutting equipment (Fig. S1 (III)). One of the sections was permanently stored in the core library of the Jilin oil field for recording and visiting, while the other was available for research to log lithological and geological information by professional geological staff. The core section was partitioned into 10–30 cm slices using a sterile rock saw, and the cutting sites were essentially determined based on lithological changes along core segments. Normally, five to ten core slices were gathered per meter core segment. The collected slices, clearly labeled with their corresponding depth, were immediately sealed in sterile, two-layer tinfoil packages. After completion, they were placed in foam cases containing dry ice and transported promptly to our laboratory for analysis. It is worth noting that on-site sampling of cores must be completed within one hour, and the exposure time of core samples for preprocessing was restricted to not exceed half an hour in order to avoid changes in physiochemical and microbiological composition.

Production water sampling and processing

The property of heavy oil renders it difficult to extract from reservoirs by the natural pressure within the reservoir. Oil/water mixtures were therefore extracted to the surface by water-flooding to recharge the pressure of the reservoir, known as artificial lifting for oil recovery. As the four wellheads were newly developed, no previous circulating formation water is available. The injection water used in water-flooding recovery is derived from local groundwater. Production fluids extracted from reservoirs to wellheads successively pass through near-well zones, perforation tunnels, wellbores interiors, and artificial lifting devices. Production fluid samples collected at wellheads were filled to the top of 500 mL sterilized boro glass bottles and tightened the caps to avoid oxygen intrusion. The oil-water mixture samples from four wells were transported to the laboratory promptly for DNA extraction, with exact sampling time and well number clearly labeled.

DNA extraction and sequencing

Extraction of genome DNA from production water

Bacterial genomic DNA in production water and oil samples was extracted with Mag-Bind® Environmental DNA Kit (Omega Bio-Tek, USA) following the manufacturer’s protocol. Cells from 80 mL PW samples were harvested by centrifugation at 13,000 × *g* for 10 mins. After the addition of lysis buffer (0.8 mL Buffer SLX Mlus), cells were resuspended by pipetting and transferred to grinding tubes containing 500 mg glass beads. DNA was released by a glass bead beating step at 6 m/s for 5 mins using the Bioprep-24 Homogenizer (ALLSHENG, China). The remaining procedure of the extraction was carried out following the instructions. DNA was purified and washed by the

means of magnetic separation using MagSi particles. The supernatant (washing solutions) was removed repeatedly, leaving DNA adsorbed to MagSi particles. Finally, the purified DNA was eluted with 80 μL elution buffer and frozen at -20°C until PCR analysis. The quality of DNA was accessed using a NanoDrop 2000 spectrophotometer (Thermo Scientific, Waltham, MA, USA). Additionally, using the same protocol, a control extraction without any sample was conducted in parallel as negative DNA extraction controls to assess the potential contamination from kit reagents or environments.

Extraction of genome DNA from sandstone cores

The extraction of gDNA from processed sandstone cores was performed using the PowerSoil DNA isolation kit (Mo Bio Laboratories, Carlsbad, USA) based on the comprehensive evaluation of DNA recovery of several extraction approaches from deep bentonite samples [8]. 0.25 g of pulverized cores, which is the classic amount of sample for this kit, was weighed and rinsed with iso-octane to remove residual heavy oil adhering to core surfaces. After centrifugation, the supernatant was discarded, and 800 μL of PowerBead buffer was added to disperse core particles and protect DNA by vortex mixing. After the addition of 60 μL lysis solution (C1 solution), cells were incubated at 60°C for 5 mins for cell lysis and DNA desorption from the core matrix. Cellular content was further released by glass bead beading at 6 m/s for five 45-second cycles. The mixing solution was then centrifuged at 10,000 × *g* for 5 mins to precipitate insoluble. Around 500 μL of DNA-containing supernatant was transferred to a clean 2 mL collection tube and washed three times with alternating C2, C3, and C4 solutions to remove contaminating organic and inorganic matter. Considering the low biomass of cores, we applied an improved DNA extraction protocol that included a three-in-one concentration step, thus representing 0.75 g of core minerals per sample gDNA extraction. Three aliquots of DNA-containing supernatants were separately extracted and mixed before passing through a silica spin filter membrane where DNA is adsorbed. Then, the DNA bound to the membrane was lastly washed with 500 μL ethanol based wash solution (C5) and centrifuged at 10,000 × *g* for 1 min. Purified DNA was eluted with 80 μL elution buffer (C6) and frozen at -20°C until PCR analysis.

Amplicon generation and sequencing

The V4–V5 regions of 16S rRNA genes were amplified using the universal primer pair 515F–907R with the barcode. All PCR reactions were performed in 30 μL reactions consisting of 15 μL of 2×Phusion® High-fidelity PCR Master Mix (Lot. M0530S, New England Biolabs), 1 μL each of forward and reverse primers, and around 10 ng template gDNA. Amplification was carried out in the following mode: initial denaturation at 98°C for 1 min, followed by 30 cycles of 98°C for 10 s, 50°C for 30 s, and 72°C for 30 s, and final elongation at 72°C for 5 mins. Two no-template controls

were included in each batch of PCR. The PCR product was quality-checked by electrophoresis on 2% agarose gel and then mixed in equidensity ratios. The mixture PCR was purified with the Universal DNA kit (TianGen, China). Sequencing libraries were generated using NEB Next® Ultra DNA Library Preparation Kit (Illumina, USA) and assessed on Agilent 5400 Fragment Analyzer System. Finally, 250 bp pair-end reads were generated by sequencing the library on an Illumina Novaseq6000 platform.

Enumeration of bacterial density in sandstone cores and PW

Enumeration of culturable aerobes and anaerobes by plate counts

Cell population of culturable bacteria in sandstone cores was determined via plate counts as previously described [8, 9], with several modifications. Two specimens from each well with a total of eight core samples were selected for cell enumeration. Core slurry was prepared by slowly adding 2 g of pulverized core powders with a moisture content of 4.52% to 18 mL 0.01M sterilized phosphate-buffered saline solution (PBS, buffered to pH 7.6) in a 50 mL agitated flask equipped with a magnetic stirring device to avoid clumping. The slurry was further dispersed by shaking vigorously on a rotary shaker for 30 mins at 30°C to mix well. 10-fold serial dilutions of the core suspension were prepared in R2A medium used for enumerations. Three dilution gradients (10^0–10^{-3}) of 100 µL core suspensions were plated in triplicates on R2A agar plates, which were incubated under oxic and anoxic conditions at 30°C for 5 and 21 days, respectively. Similar operations were performed in diluents (sterilized R2A medium) as blank controls to assess environmental contamination. Enumeration of cell abundance in PW follows similar procedure. PW was serially diluted (10^{-2}–10^{-5}) in R2A medium and 100 µL of diluted samples were inoculated in R2A agar plates.

Enumeration of bacteria by quantitative PCR

To ensure an accurate estimation of bacterial abundance, genomic DNA was quantified by targeting multicopy bacterial 16S rRNA genes using primers 27F/338R on the platform of Bio-Rad MYiQ2 real-time fluorescence quantitative PCR. The PCR system is composed of a 20-µL reaction mixture with 10 µl 2×RealStar Green Fast Mixture (GenStar), 0.4 µL forward and reverse primers, and 40 ng (1 µL) DNA template. The qPCR runs were performed under the following conditions: 95°C for 5 mins followed by 40 cycles of 95°C for 10 s, 50°C for 30 s, 72°C for 30 s, and 80°C for 10 s. Amplification was performed in triplicates by virtue of the 96-well optical reaction plate, and three negative PCR controls using nucleic acid-free PCR water in place of DNA templates were included for each batch of qPCR.

We employed *Escherichia coli* K-12 genomic DNA as the internal reference for the absolute quantification of cDNA. *E. coli* K-12 was cultured overnight in 100 mL LB medium at 37°C and

serially diluted 10-fold from 10^{-1} to 10^{-8}. At this moment, Cell density was quantified to be 8.21× 10^9 by plate counts of various diluents of 10^{-6} to 10^{-8}, and simultaneously the genomic *E. coli* DNA was extracted from the sample volume (100 μL) of 10^0 to 10^{-4} diluents. DNA quality and concentration were assessed using a NanoDrop 2000 spectrophotometer. The concentrations of the DNA recovered from 10^{-1} and 10^{-2} diluents were proportional, suggesting a high DNA recovery efficiency within the concentration range. Therefore, we used the DNA extracted from 10^{-1} *E. coli* bacterial suspension as the standard internal reference for subsequent qPCR analyses. For each qPCR run, five 10-fold dilution gradients of *E. coli* K-12 genomic DNA from 10^{-1} to 10^{-5} were performed in triplicates. Standard fitting curves were drawn by plotting threshold cycle (C_T) against known standard bacterial concentrations. The qPCR results from triplicate samples were calculated and averaged from the fitting curve. The bacterial abundance was finally expressed in terms of CFU per mL of production water after normalizing the volume of PW (80 mL) used for DNA extraction.

Supplementary Results

Cell densities in petroleum reservoir environments

The average numbers of cultivated bacteria in sandstone core samples were 9.39×10^6 and 5.14×10^5 CFU per gram of dry weight, respectively (Table S5), which are much less than natural soils and sediments (ranging from 10^8 to 10^{10}) [10, 11], but higher than highly compacted bentonite in deep geological repository (10^2~10^5) [8, 12, 13]. The absolute bacterial biomass in PW quantified by qPCR was $(6.31 \pm 0.81) \times 10^6$ CFU mL^{-1}, whereas the cultivated aerobic and anaerobic bacteria determined by plate counts averaged $(2.13 \pm 0.4) \times 10^5$ and $(1.76 \pm 0.88) \times 10^4$ CFU mL^{-1}, which are comparable to the previously reported cell density of production fluids in petroleum reservoirs (average: 5.84×10^5 CFU mL^{-1}) [14]. Cell abundance estimated by quantitative PCR was higher than that with the cultivation-based approaches since a large number of microbes are uncultured or cannot grow in the specific culture medium or conditions. Additionally, extracellular DNA could be recovered via qPCR, resulting in an overestimation. Therefore, we quantified bacterial densities using both qPCR and culture-dependent methods to comprehensively assess cell densities.

When examining the dynamic variation of bacterial populations, it is noteworthy that bacterial cell density in PW was significantly enhanced in the short term after the injection of exogenous bacteria, and then returned to its initial level gradually (Fig. S13c). This is in accordance with the recognized laws that the addition of exogenous bacteria can lead to a transient rise in bacterial biomass; however, cell density will eventually maintain a relatively stable level due to the limited energy and carbon sources and fierce intra- and inter-species competitions in reservoirs.

Hidden anoxic sulfur and nitrogen cycles for electron acceptor generation

Although the N/S elements in heterocycles are released in dissolved reduced forms, significant regeneration of sulfate and nitrate was observed after the microbial modulation, implying that there must be hidden anoxic resupply of sulfate, nitrate, and/or their intermediate variants to fuel the cryptic sulfur and nitrogen cycles in the subsurface reservoir. The oxidation mechanism could be intricate due to the diverse microorganisms and geological components of petroleum reservoirs. We concluded that microbial and redox-active matter-mediated (biotic and abiotic) oxidation processes collectively sustain the oxidation of reduced sulfur and nitrogen from heterocycle degradation, assuring continued sulfite/sulfate and nitrite/nitrate production.

To determine the role and contribution of microbial oxidation of reduced sulfur in sulfate production, we investigated sulfur oxidation pathways and relevant genes (Fig. S15a). The marker genes encoding enzymes for the oxidation of reduced form of sulfur compounds (H_2S, S^0, $S_2O_3^{2-}$, and SO_3^{2-}) include sulfide quinone oxidoreductase (*sqr*), catalyzing the oxidation of H_2S to

elemental S^0, reverse-acting dissimilatory sulfite reductase (*rdsr*) for oxidation of S^0 to SO_3^{2-}, thiosulfate sulfurtransferase (*TST*, *glpE*) for oxidation of $S_2O_3^{2-}$ to SO_3^{2-}, SOX complex system (*sox*) for oxidation of $S_2O_3^{2-}$ to SO_4^{2-}, and adenosine 5'-phosphosulfate reductase (*apr*) and sulfate adenylyltransferase (*sat*) for oxidation of SO_3^{2-} to SO_4^{2-}. We found that the microbiomes of PW in August possessed the complete suite of genes for the oxidation of reduced sulfur compounds to sulfate, consistent with the rapidly increasing sulfate concentrations and the predominance of sulfate-reducing bacteria. Particularly, *rdsr* mediating the oxidation of S^0 to SO_3^{2-} was significantly enriched in PW (8). It is a critical step for sulfate production and presumably accounts for sulfate accumulation in PW of August. Previous studies also indicated that microbial oxidation of reduced sulfur compounds was pervasive and an essential source of free energy for the growth of specific groups of sulfur-oxidizing *Gammaproteobacteria* in the deep sea, where these electron donors are enriched [15].

An alternative explanation that underpins a major flux from sulfide to sulfate is oxidation mediated by redox-active chemicals like humic and fulvic acids, metallic oxides (Fig. S15b). In anaerobic petroleum reservoirs, multiple metallic oxides (e.g., iron minerals, MnO_2, TiO_2, MgO) might implicate in N/S cycling. There is cumulative evidence that redox-active matter fuels the first step of sulfide oxidation to either elemental sulfur or thiosulfate. Recent studies revealed that sulfur cycling was tightly coupled with iron cycling, and the sulfur-fueled iron reduction is a dominant pathway for producing sulfate and reducing iron oxides [16], which accounts for the unexpectedly high rates of sulfate reduction and active sulfur respiration in low-sulfate anoxic ecosystems, such as wetland and ocean sediments. Additionally, the quinones of humic acids and other dissolved organic matter (DOM), as major redox-active functional moieties due to the exceptional electron-accepting capacity, could also mediate the chemical oxidation of hydrogen sulfide to thiosulfate [17, 18], suggesting that natural organic matter might be the key to anoxic sulfur oxidation. Intermediate sulfur compounds can either be reduced back to sulfide, disproportionated to sulfate and sulfide, or completely oxidized to sulfate if in the presence of Fe (III) and Mn (IV) [19], resulting in the replenishment of sulfate in the reservoir.

Reduced forms of nitrogen such as ammonium (NH_4^+) can be partially oxidized to nitrite or nitrate by ammonia-oxidizing bacteria (AOB) or ammonia-oxidizing archaea (AOA) in hypoxic environments. Oil production processes are inevitably accompanied by minimal dissolved oxygen (DO) ingression due to the influx of oxygenated, precipitation-derived (meteoric) waters and water and inoculum injection. Moreover, subsurface bioturbation was regarded to deliver oxygen intermittently that sustains nitrification even below the typical redox gradient in sediments, which explains the active ammonium oxidation and presence of AOB/AOA in sediments where oxygen

was essentially undetectable [20]. AOA are far more abundant and adaptable to extreme environments than AOB in subsurface environments [21, 22]. Our archaeal analysis revealed that the two typical groups of AOA, family *Nitrososphaeraceae* and *Nitrosopumilaceae*, were abundant in all reservoir samples, comprising averaged 36.0% and 0.176% of the total archaea (Fig. S5). Therefore, we argued that microbial ammonia oxidation through AOA/AOB could be important in such hypoxic petroleum reservoirs, resulting in nitrate/nitrite production.

Additionally, anaerobic ammonium oxidation is coupled with iron reduction, which has been discovered to commonly occur in iron-rich environments, referred to as ferric ammonium oxidation (Feammox) process [23, 24]. The stoichiometry and free energy change of Feammox with the most common insoluble iron oxides serving as electron acceptors were shown below (Eq. 1). The evident negative Gibbs free energy change indicates the reaction is thermodynamically favorable. The group of *Actinobacteria* was demonstrated to play a key role in the simultaneous ammonia oxidation and iron reduction [22], which was highly enriched in our lithospheric minerals. The geochemical measures of sandstone cores also indicated that iron minerals (ferrihydrite, 1–5%) are abundant in the petroleum reservoir. Therefore, iron (III) mineral mediated ammonia oxidation could potentially be one of the contributors to nitrate/nitrite production.

$$3Fe_2O_3 \cdot 0.5H_2O + 10H^+ + NH_4^+ \rightarrow 6Fe^{2+} + 8.5H_2O + NO_2^-,\ \Delta G_r \leq -145.08 \text{ kJ mol}^{-1} \qquad \text{(Equation 1)}$$

In summary, there is mounting evidence and theories collaborating the active nitrogen and sulfur oxidation that fuels the cryptic elemental cycles in anoxic marine and terrestrial sediments. However, more experimental validation of these plausible explanations and identification of the major contributors to recycling of reduced sulfur and nitrogen to sulfate and nitrate that sustains the observed high reduction rates are still required.

Assessment and analysis of the biostimulation performance on oil recovery

According to the field results, the productive effectiveness of microbial modulation on wells #1 and #4 were observed and validated; however, the effect on wells #2 and #3 was not as pronounced, for several reasons. It is worth mentioning that the reservoir's subsurface environment is highly heterogeneous, with physiochemical and geological compositions that can significantly differ even within one well pattern. It was also demonstrated by our geochemical measurements of core samples, which revealed that permeability, oil and water contents varied from well to well, and even within the same well as depth increased (Supplementary Dataset S1). Especially for well #2, the heterogeneity of the stratum is obvious (the permeability is 2,998 mD at the depth of 192.3m, but it changed to 881 mD at 205.5m depth), leading that water and bacterial inoculum primarily enter high

permeability sections with low pressure and less remaining oil. The low vertical sweep efficiency (fraction of a formation in the vertical plane with which water will contact) deteriorates the microbial stimulation and oil displacement efficiency for well #2. On this basis, it is understandable that microbial enhanced oil recovery (MEOR) techniques cannot always achieve desirable results for all wells; however, the analysis of the problems and the discussion of applicability are paramount for the popularization of the technology.

In this practice, unlike wells #1 and #4, wells #2 and #3 were shut down on August 17th and reopened on September 27th due to production and safety concerns. For well #2, the reservoir hydrogen sulfide and sulfate concentrations still remained at a very low level (5 ppm) until August. We speculated that the flux of S release, controlled by the heterocycle degradation, was insufficient, so we shut down well #2 for a while to promote N,S-heterocycle degradation by prolonging microbial interaction. Eventually, the sulfide content in well #2 increased to around 80 ppm, and sulfate was also detectable in mid-October, but still less than other wells. However, well #3 encountered a completely different situation, with a sulfide concentration as high as 708 ppm after reopening for production. Such high sulfide concentration will pose serious health risks to personnel and complicates downstream refining operations. To maintain safety, we shut down well #3 on August 17th, awaiting the reduction of hazardous hydrogen sulfide to an acceptable level by sulfide oxidation. Consequently, the oil recovery efficiency of wells #2 and #3 was inferior to that of wells #1 and #4.

The geological condition of the reservoirs is the key to heavy oil production no matter for conventional oil production or our MEOR approaches. We always expect the reservoir is homogeneous, but in reality rocks exhibit a range of pore sizes which causes a variation in capillary pressure and permeability. Aiming at this kind of issue (like Well #2), we propose to inject temporary plugging agents, typically hydrolyzed polyacrylamide (HPAM), to plug the high permeability regions (large pores) and ameliorate the stratum heterogeneity before employing our MEOR approach.

Assessment on effects of reagent and laboratory contamination

We followed the “RIDE” checklist for biomass studies [25], and included a total of three DNA extraction blank controls and three DNA no-template amplification controls in three batches of high-throughput amplicon sequencing. The extracted DNA concentrations of blank controls in three batches were measured to be less than 4 ng/μL (NanoDrop 2000 spectrophotometer). Despite no

visible amplification bands detected by gel electrophoresis, they were sent for library preparation and sequencing. We also included no-template amplification controls during PCR library preparation in order to distinguish potential contaminations from reagents and the laboratory environment during library preparation and sequencing.

A total of three DNA extraction kit controls and three no-template controls were included. The β–diversity analyses of controls and samples showed that whether NTCs or KitCtrls were clearly separated from our biological samples (Fig. S18a), indicating an evident difference in community structure between controls and biological samples and, thus, negligible impacts on our biological samples. The taxonomic profiles of controls revealed the potential contaminant taxa, as shown in Fig. S18b. No common taxa were identified in all controls and there were few overlapped taxa with biological samples. Most reads were associated with common reagent and laboratory contaminants, such as *Methylobacterium*, *Chryseobacterium*, and *Pseudomonas* [25]. However, true reads, especially those affiliated with high-abundance ASVs, are inevitably present in part of negative controls due to the demultiplexing error or cross-contamination of samples, which must be considered in the analysis as long as they are not predominating. In addition, the very low DNA concentration, the low read counts (hundreds of reads in controls versus >30,000 reads in samples), and the randomized taxonomic proportions among controls suggest that the error rate and contamination are nuanced and negligible. Especially, the significant presence of *Acinetobacter*, *Burkholderiaceae* and *Dietzia* found in low-biomass sandcore samples were not detected in all controls. As a result, the microbial communities of PW and sandstone cores we reported were less likely to be impacted by contaminant taxa, but reflects the real microbiome.

Additionally, we used sterilized instruments in a clean laminar-flow hood where surfaces and equipment were frequently disinfected with bleach and UV radiation if not in use to minimize contaminant DNA for most sample processing operations like aliquot and packaging of PW and cores and DNA extraction. Furthermore, we have collected and sequenced a considerable repertoire of representative PW (n = 143) and sandstone core (n = 24) samples, including many duplicates of the same moments and wells (e.g., 2-005-3 and 2-005-4, 4-001 and 4-001-2) with a high degree of repeatability.

Supplementary Figures

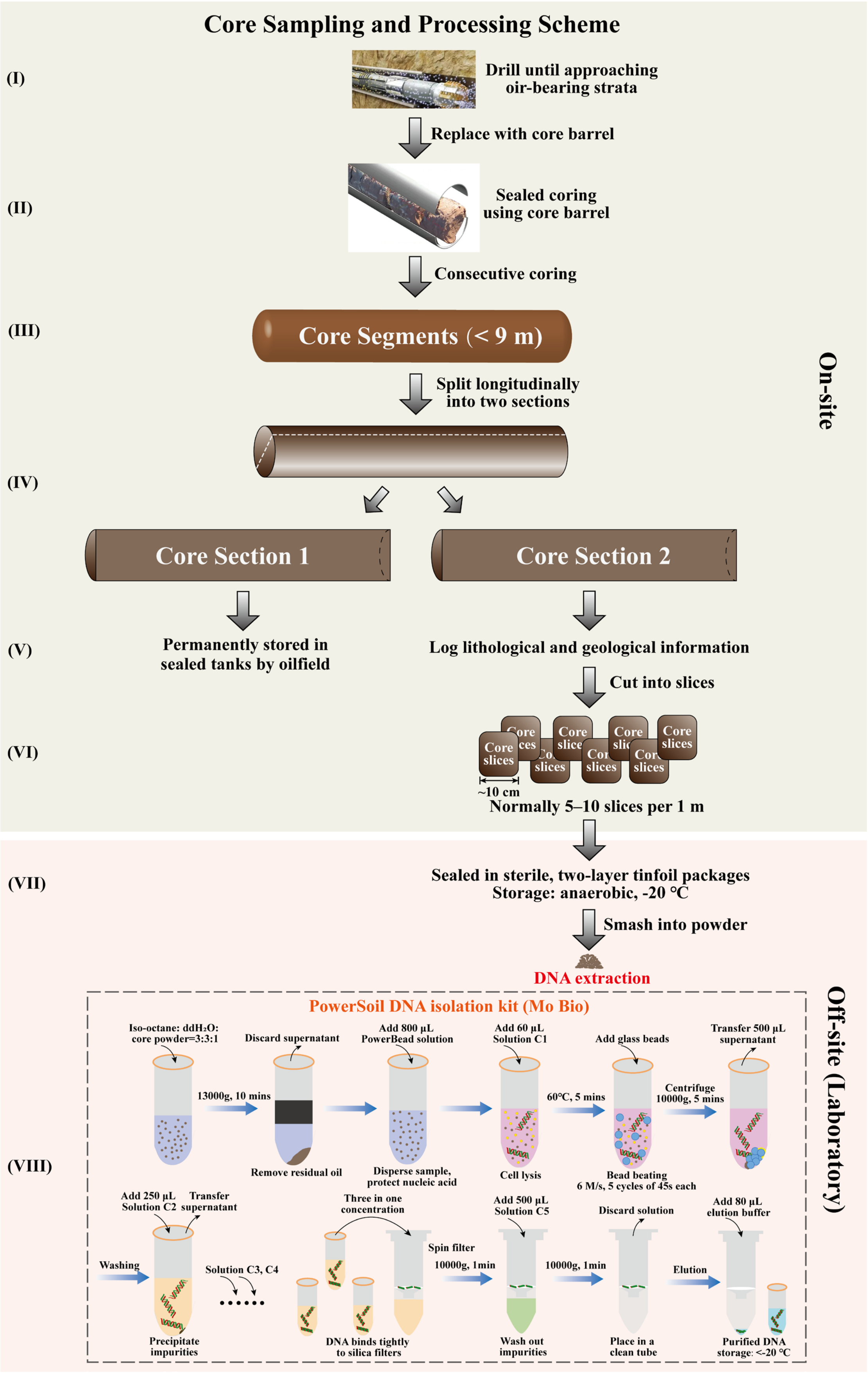

Supplementary Fig. S1. Schematic diagram of sandstone core sampling and processing for microbiome analysis. Core samples were retrieved at depth of approximately 185-205.5m. (I) Drilling operations using a rock bit until approaching oil-bearing reservoirs according to the previous local geological survey. (II) Lowering coring barrel for sealed coring. (III) Consecutive coring collects core segments until approaching the base of the oil column. (IV) Splitting core segments into two core sections along vertical profiles using special cutting machines. (V) One section was permanently backed up by the oilfield for recording and visiting, and the other was measured and studied to log lithological and geological information by professional geological staff. (VI) Core sections were partitioned into core slices, and the cutting sites were determined according to lithological changes along core segments. Normally, five to ten core slices were collected per meter core segment. (VII) The collected slices were immediately sealed in sterile, two-layer tinfoil containers, which were put in foam boxes filled with ice, and immediately transported to our laboratory for storage (anaerobic, -20℃). Core slices were prepared for DNA extraction by smashing into powders. (VIII) Improved DNA extraction protocol for low-biomass core samples using PowerSoil DNA isolation kit.

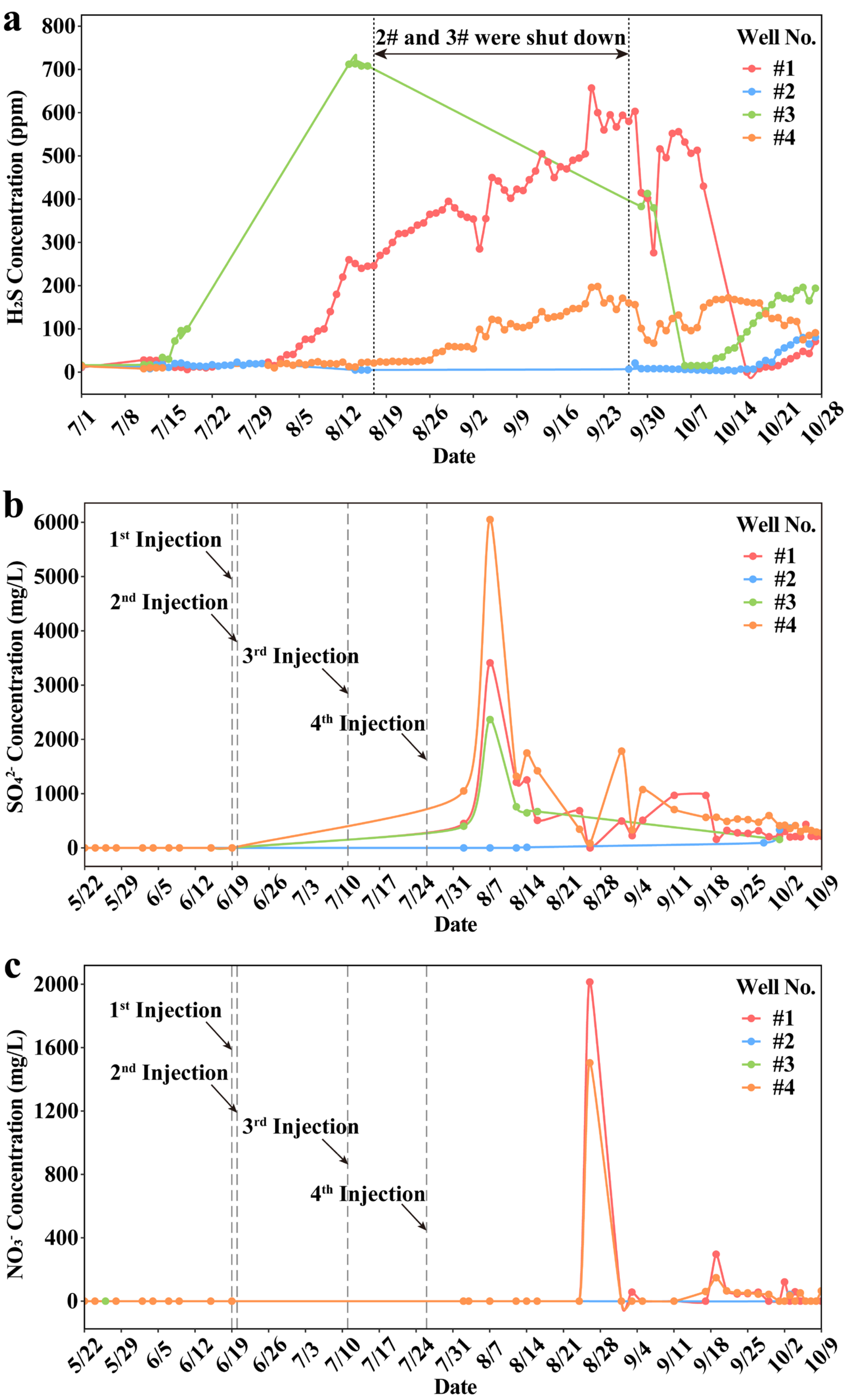

Supplementary Fig. S2. The temporal variations in major chemical forms of sulfur and nitrogen during the entire period of field trials. (a) variations of hydrogen sulfide concentration over time in petroleum reservoirs. (b) variations of sulfate concentration over time in PW. (c) variations of nitrate concentration over time in PW.

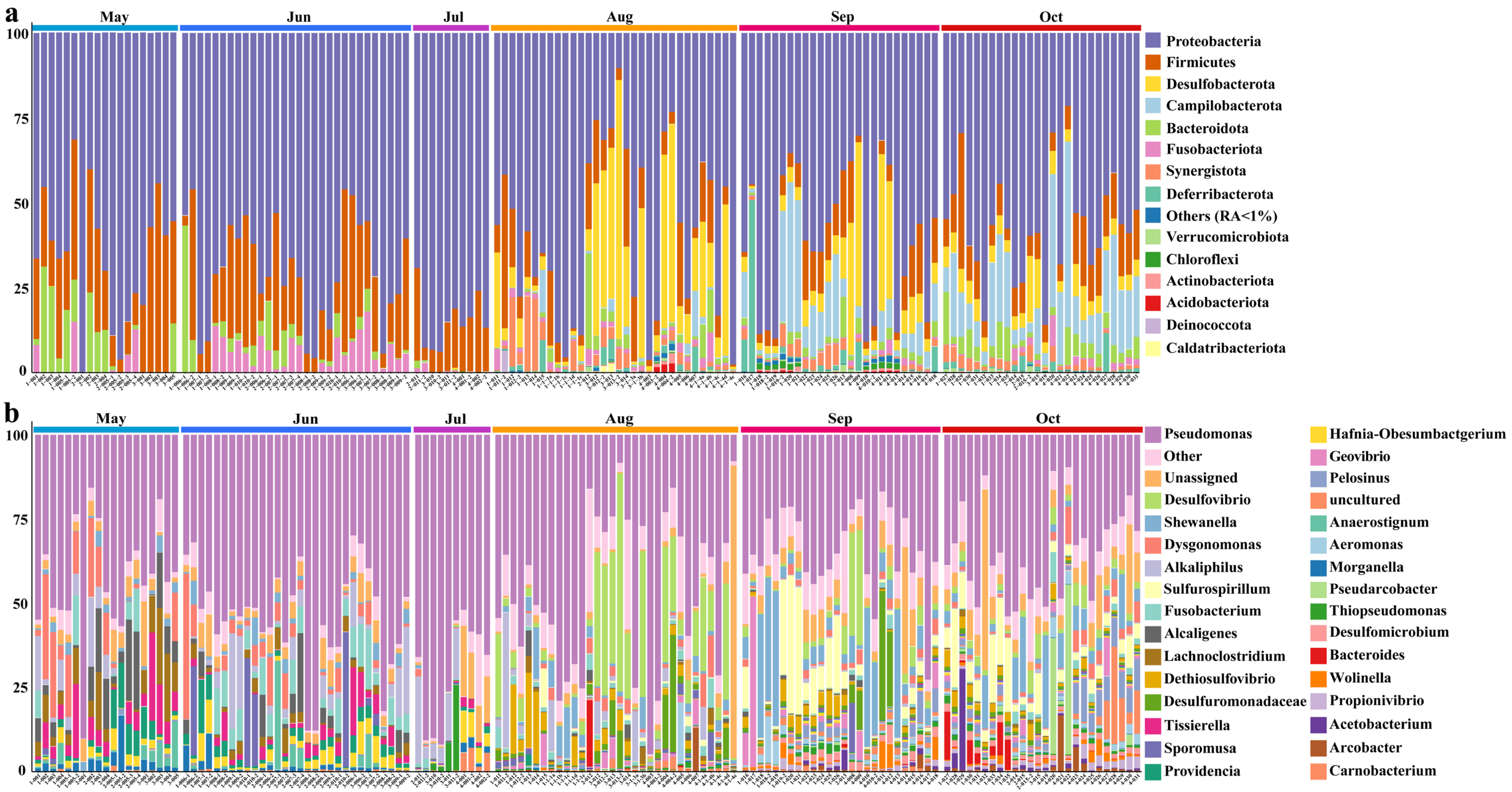

327 **Supplementary Fig. S3. The temporal variations of bacterial compositions across all PW samples (*n* = 143).** (a) at the phylum level. (b) at the genus level.

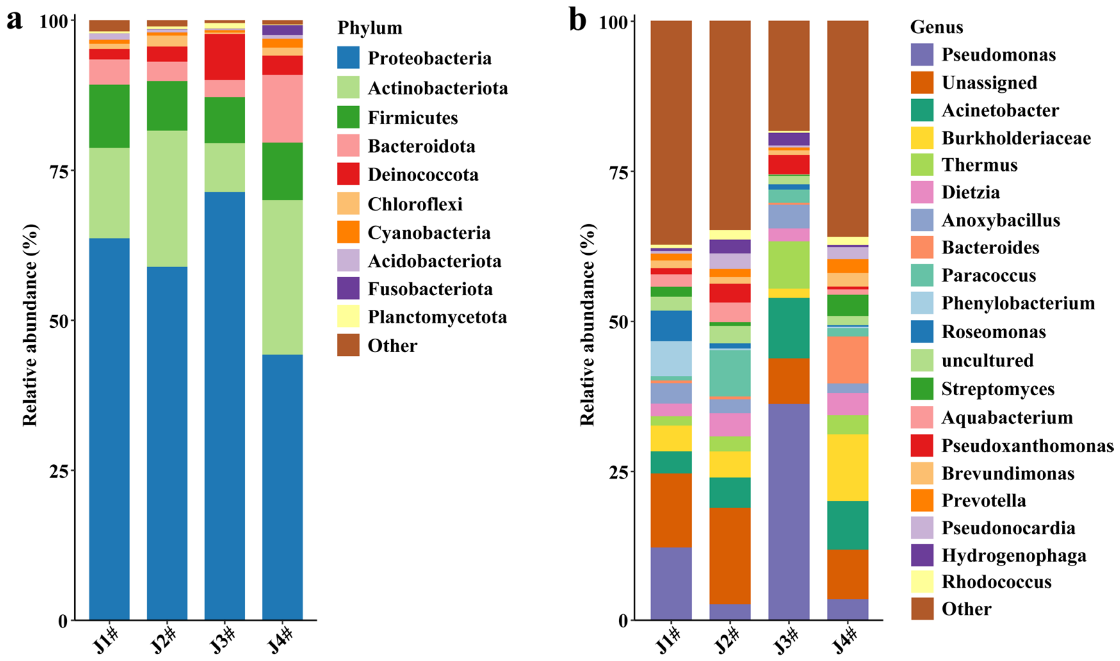

Supplementary Fig. S4. The bacterial compositions of sandstone cores across different wells. (a) at the phylum level. (b) at the genus level.

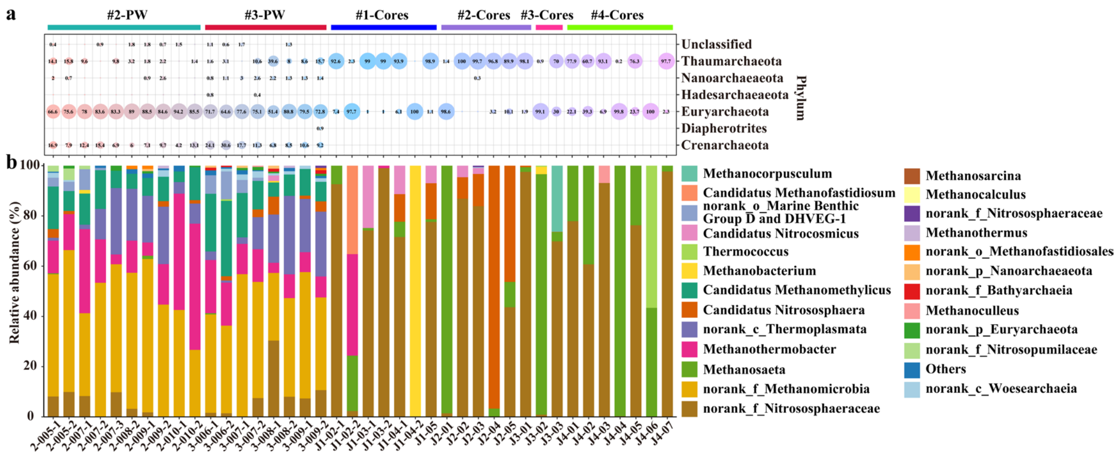

Supplementary Fig. S5. The Archaeal compositions of partial PW and sandstone core samples recovered from four wells. (a) bubble plot showing the archaeal composition at the phylum level. (b) stacked bar chart displaying the archaeal composition at the genus or family levels.

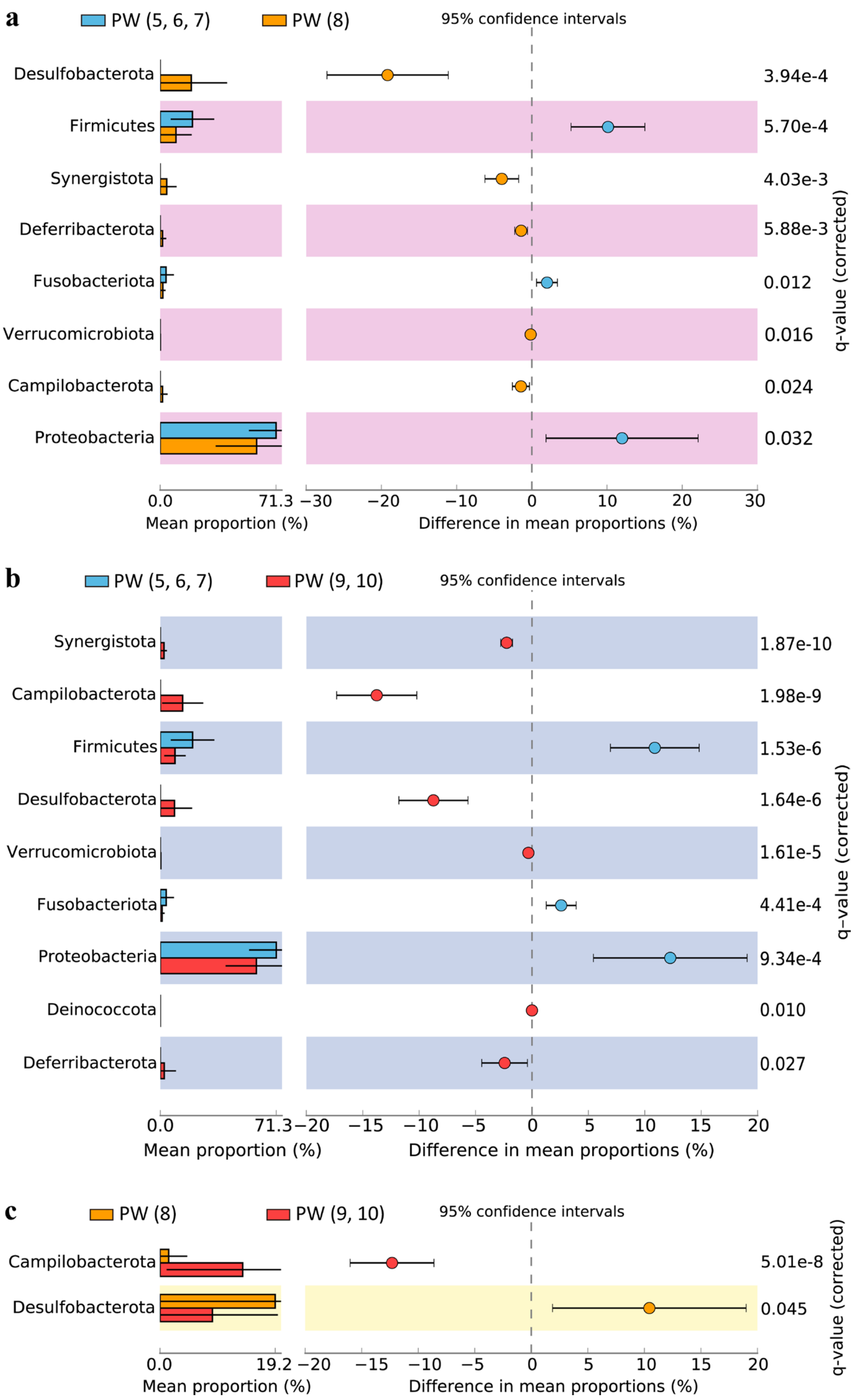

334 **Supplementary Fig. S6. Pairwise comparisons of significantly different phyla among various**
335 **stages of PW.** The *q-value* is based on Weltch's *t*-test corrected by Benjamini–Hochberg FDR.

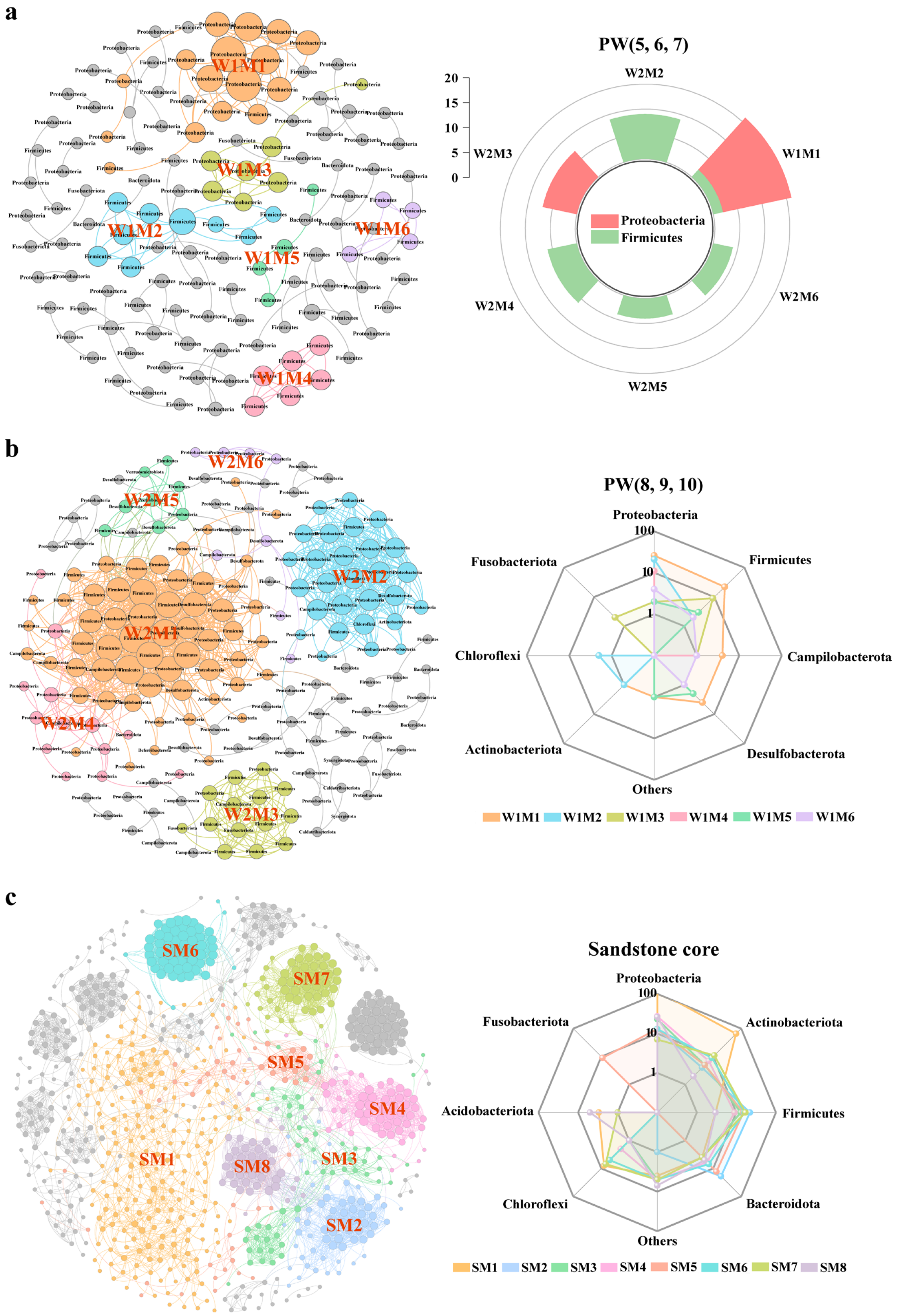

Supplementary Fig. S7. Co-occurrence subnetworks in various habitats and the node composition in each module reveal the ecological characteristics of microbial networks and environment-specific bacterial co-occurrence relationships. (a) subnetwork of PW (5, 6, 7). (b) subnetwork of PW (8, 9, 10). (c) subnetwork of sandstone cores.

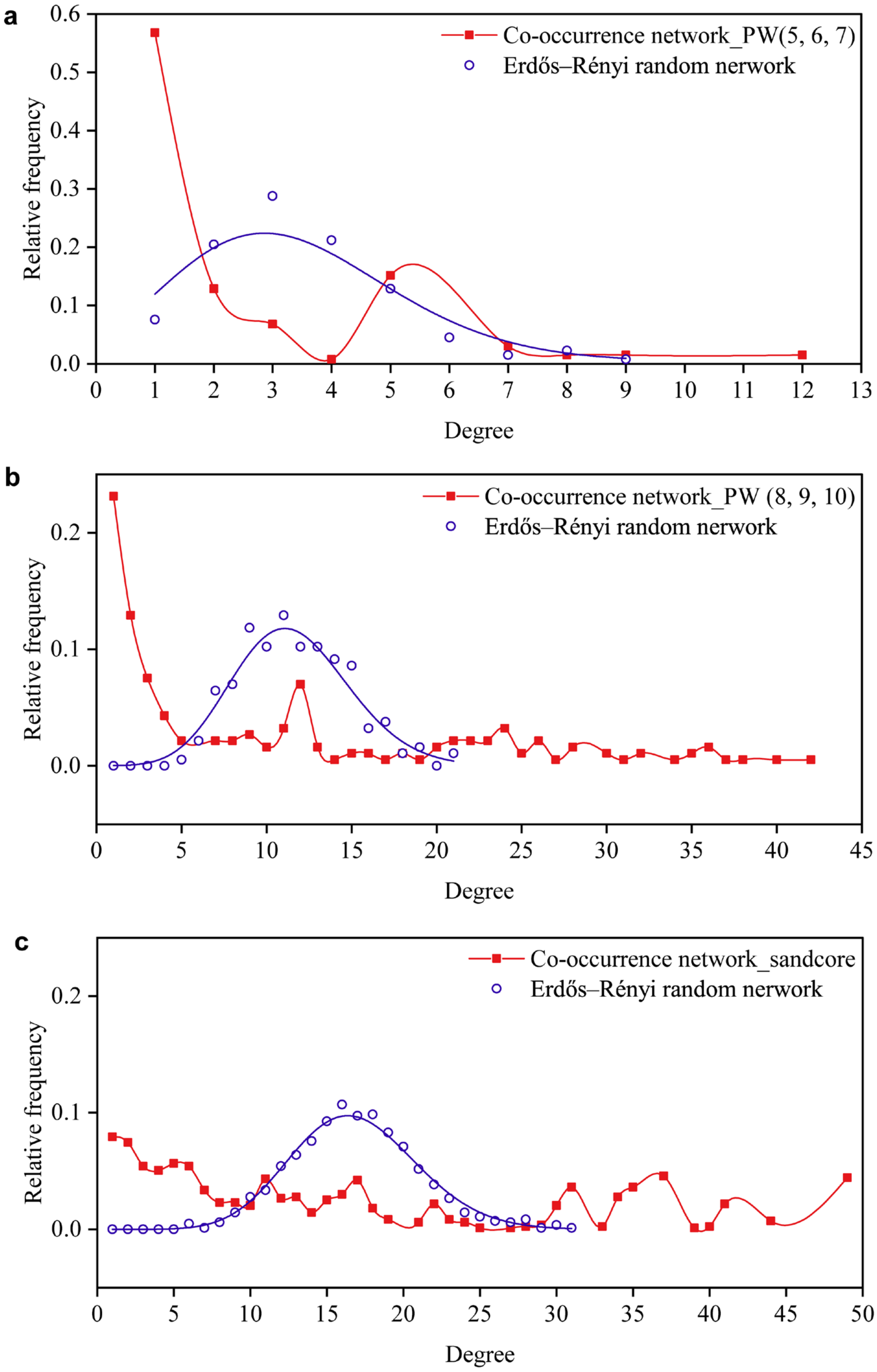

Supplementary Fig. S8. The discrepancies of degree distributions between subnetworks and their corresponding random networks. Erdős–Rényi random networks were established based on the corresponding number of nodes and edges. The generated random networks tend to be well fitted with Poison distribution, whereas the real network follows a power-law distribution. The degree distribution dissimilarity reflects the non-randomness of constructed subnetworks in our studies.

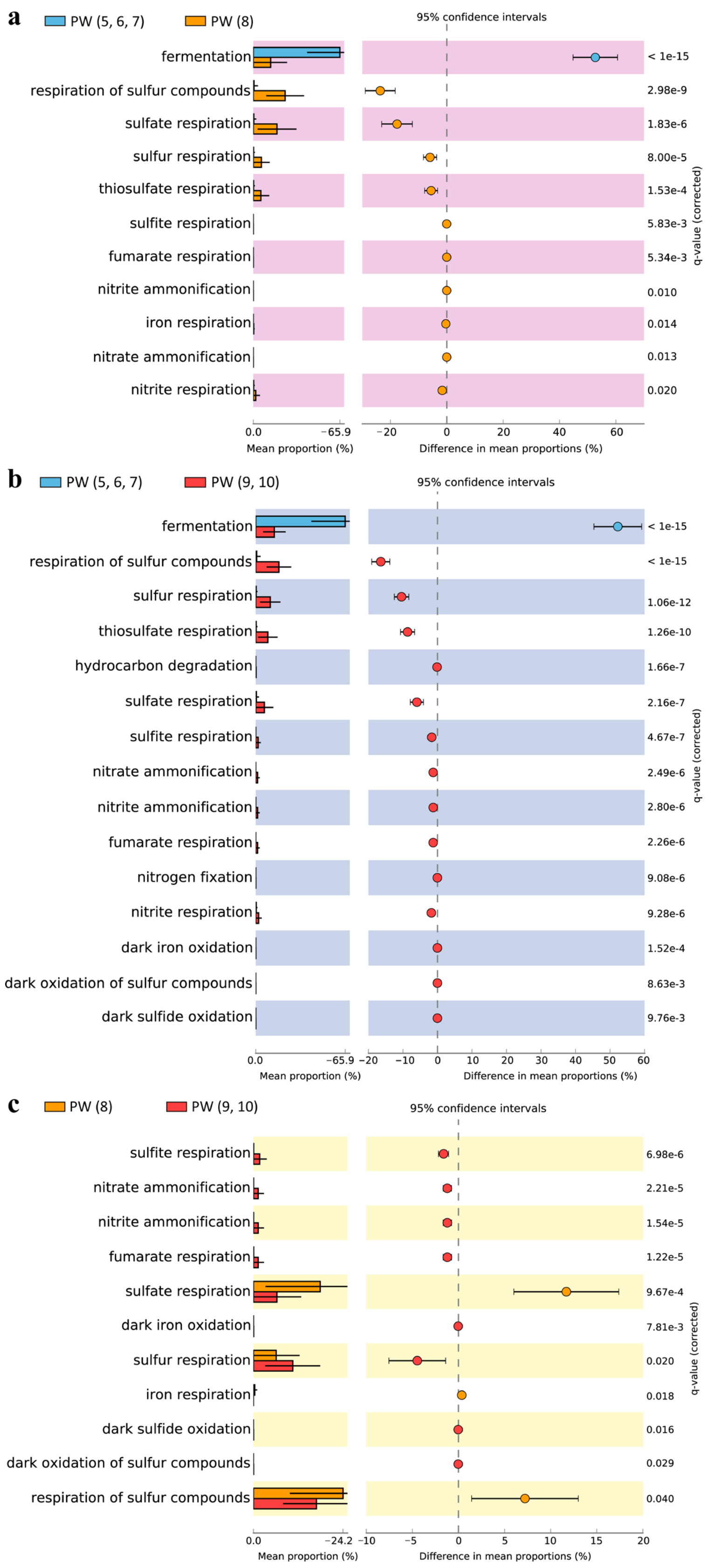

Supplementary Fig. S9. Pairwise comparisons of significantly different functions among various stages of PW's microbiomes predicted by aligning to the FAPROTAX database (FDR adjusted *p-value*< 0.05, Welch's t-test).

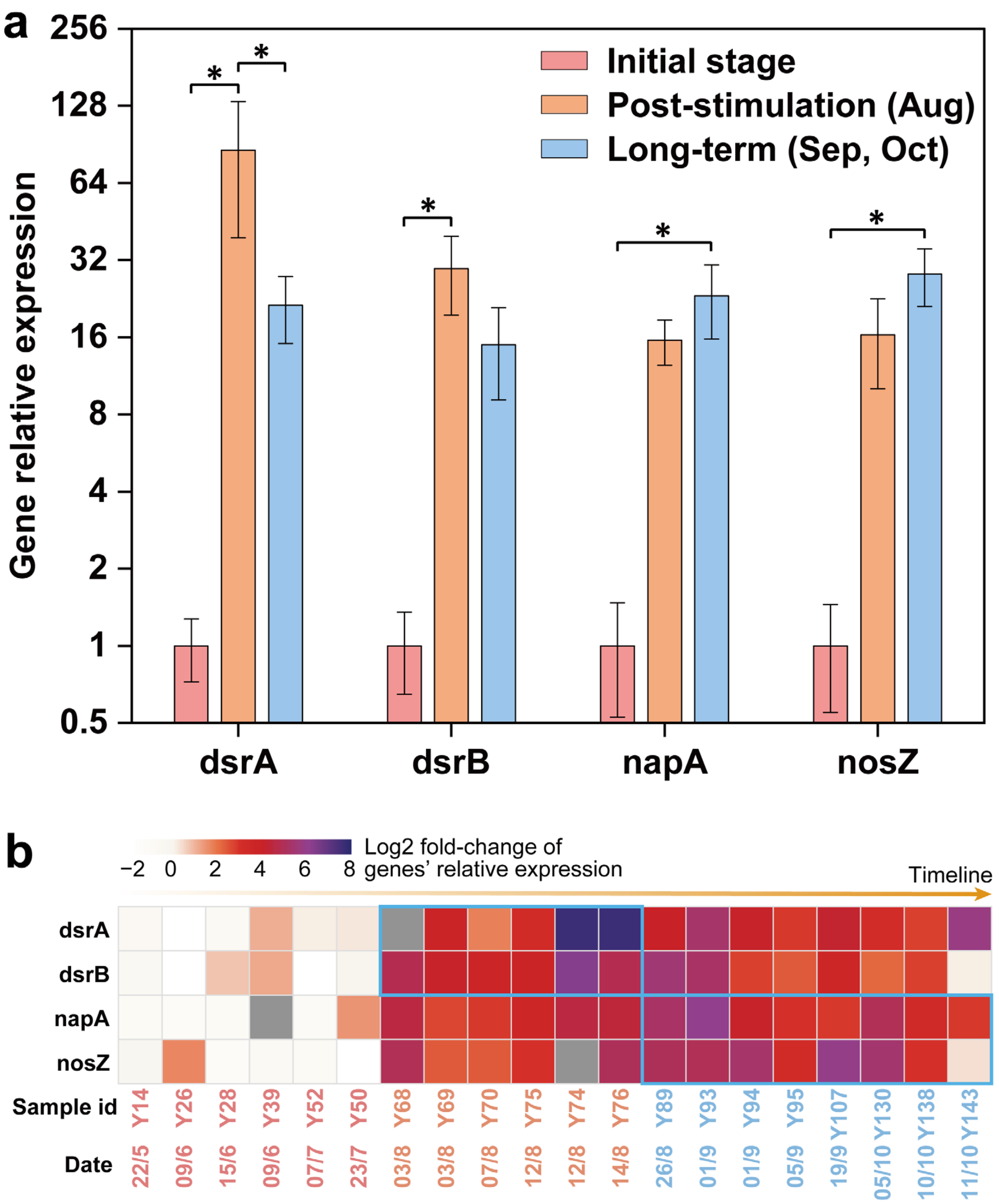

Supplementary Fig. S10. Fold-change of key genes' relative expression by RT-qPCR. (a) The average value of gene relative expression abundance at three stages. The fold-change in gene relative expression, calculated by dividing the expression level by the averaged gene expression value of the initial reservoir samples, was significantly increased after exogenous bacterial intervention. (*$P < 0.05$, One-way ANOVA with Fisher's LSD post-hoc test). (b) Log2 fold-change of genes' relative expression across time.

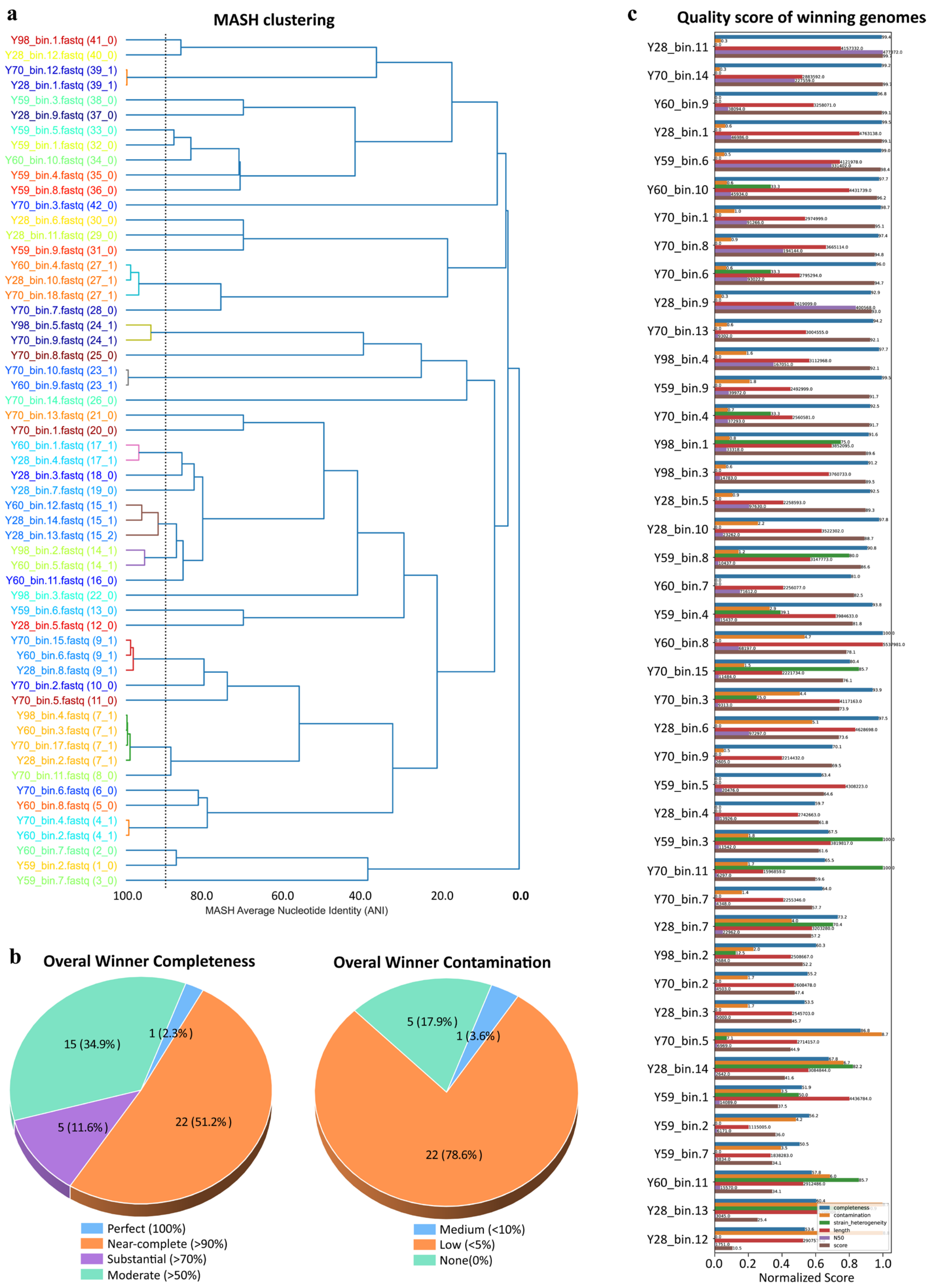

Supplementary Fig. S11. Summary of recovered MAGs. (a) Clustering results of MAGs based on mash algorithm. A total of 58 MAGs from all samples were clustered into 43 dissimilar MAGs at 90% ANI level. (b) The proportion of MAGs with different degrees of completion and contamination. (c) The detailed quality information of each MAGs.

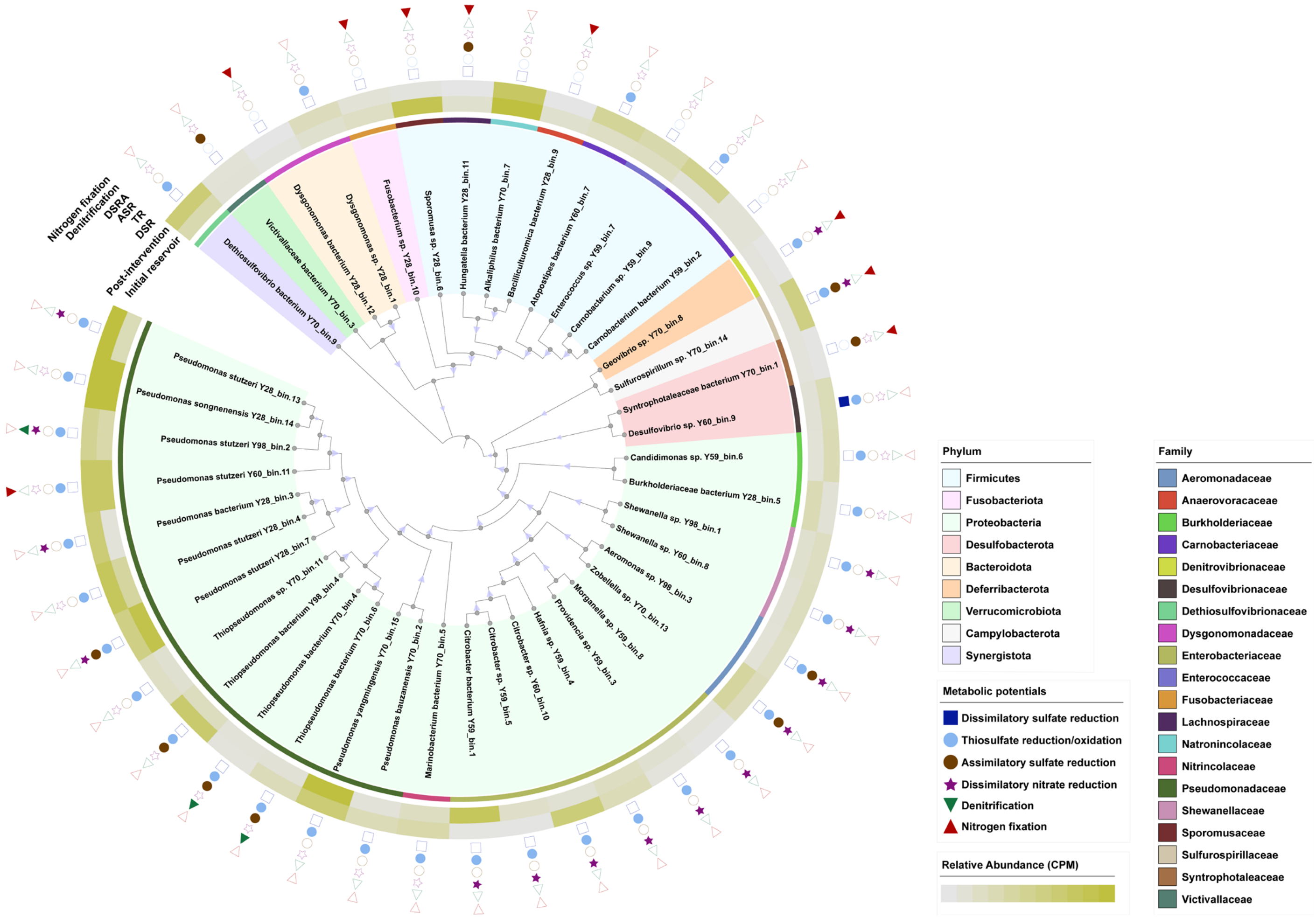

Supplementary Fig. S12. Taxonomy and phylogenetic tree of high-quality MAGs. Different colors in the inner ring represent various phyla and families. The heatmaps of the middle rings represent the genomic abundance of MAGs in the initial and post-intervention reservoir microbiomes, expressed as genome copies per million read. Nitrogen- and sulfur-cycling metabolic pathways were shown by various shapes and the presence of a complete pathway was denoted as a filled shape.

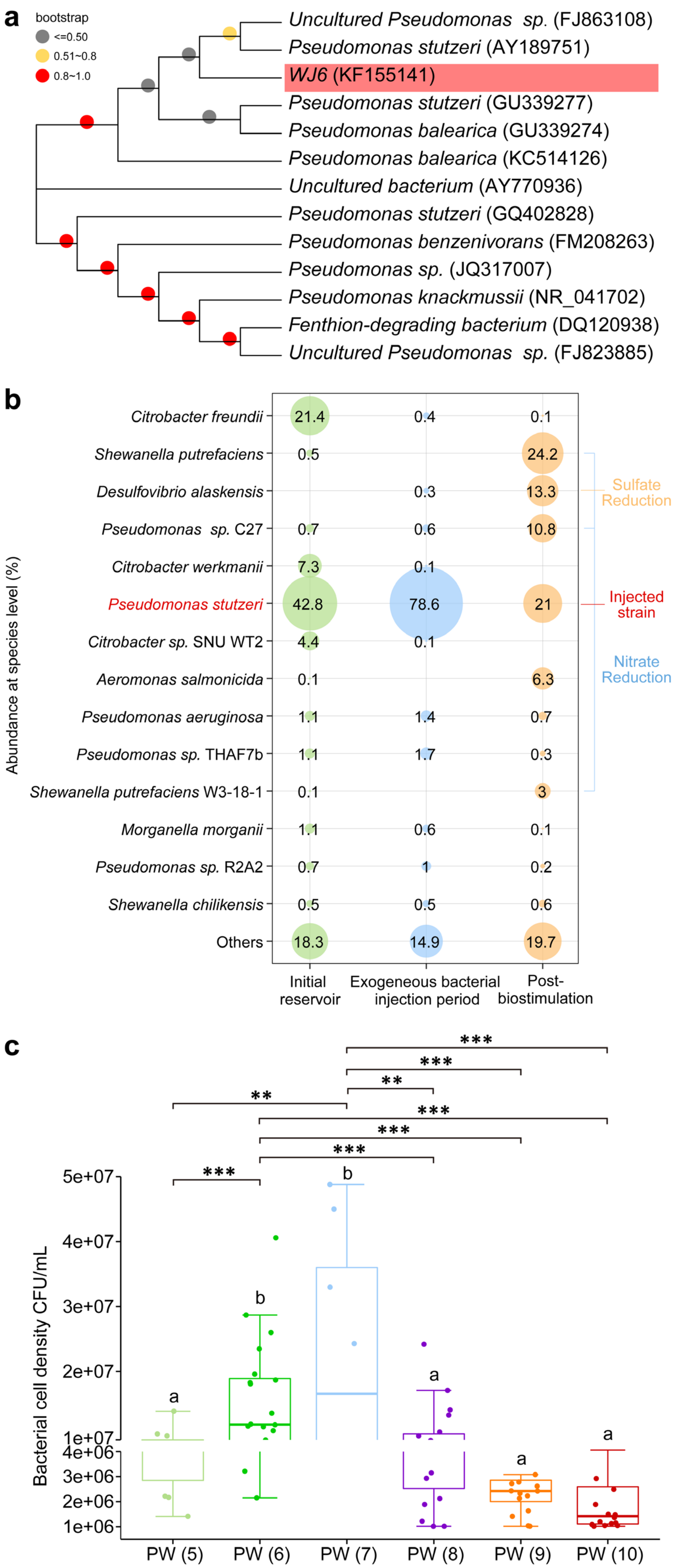

a
bootstrap
<=0.50
0.51~0.8
0.8~1.0
Uncultured Pseudomonas sp. (FJ863108)
Pseudomonas stutzeri (AY189751)
WJ6 (KF155141)
Pseudomonas stutzeri (GU339277)
Pseudomonas balearica (GU339274)
Pseudomonas balearica (KC514126)
Uncultured bacterium (AY770936)
Pseudomonas stutzeri (GQ402828)
Pseudomonas benzenivorans (FM208263)
Pseudomonas sp. (JQ317007)
Pseudomonas knackmussii (NR_041702)
Fenthion-degrading bacterium (DQ120938)
Uncultured Pseudomonas sp. (FJ823885)
b
Abundance at species level (%)
Citrobacter freundii
Shewanella putrefaciens
Desulfovibrio alaskensis
Pseudomonas sp. C27
Citrobacter werkmanii
Pseudomonas stutzeri
Citrobacter sp. SNU WT2
Aeromonas salmonicida
Pseudomonas aeruginosa
Pseudomonas sp. THAF7b
Shewanella putrefaciens W3-18-1
Morganella morganii
Pseudomonas sp. R2A2
Shewanella chilikensis
Others
Sulfate Reduction
Injected strain
Nitrate Reduction
Initial reservoir
Exogeneous bacterial injection period
Post-biostimulation
c
Bacterial cell density CFU/mL
PW (5)
PW (6)
PW (7)
PW (8)
PW (9)
PW (10)

Supplementary Fig. S13. Tracking of the variations of key functional strains and bacterial cell density in PW along the microbial modulation. (a) The phylogenetic relationship based on 16S rRNA gene sequences between strain WJ-6 and other *Pseudomonas* species as determined by neighbor-joining algorithm and evaluated by the maximum likelihood and maximum parsimony algorithms. WJ-6 shares 99.1% sequence identity to the *Pseudomonas stutzeri* species, indicating that the strain will be attributed to *Pseudomonas stutzeri* species in metagenomic result. (b) Variations of the taxonomic compositions at species level from metagenomic analysis, calculated by butt-joint of annotated genes and their best-hits in the micro-NT database. (c) Variations of bacterial cell density in PW by qPCR. The injection operation was consistent with the cell abundance change, resulting in a significant increase during late June and July and subsequently a rapid decline to the initial bacterial concentration (adjusted $^{**}P < 0.01$, $^{***}P < 0.001$, nonparametric Kruskal-Wallis rank-sum test after verifying the non-normal distribution).

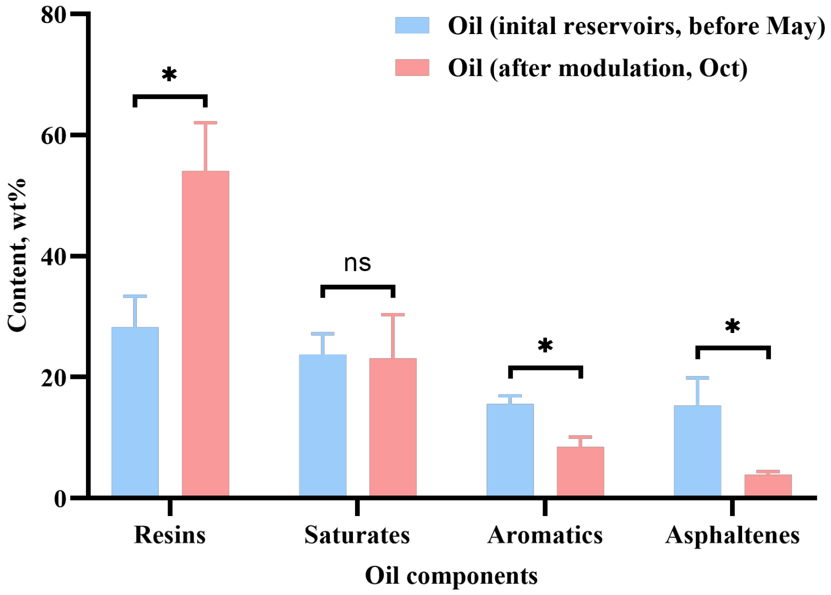

Supplementary Fig. S14. SARA analysis of heavy oil component changes from field trails by our exogenous bacterial modulation. The relative contents of aromatics and asphaltenes in heavy oil were significantly decreased ($P = 0.016$ for aromatics; $P = 0.048$ for asphaltenes, two-sided *t*-test after verifying conformity to normal distribution and uniformity of variance).

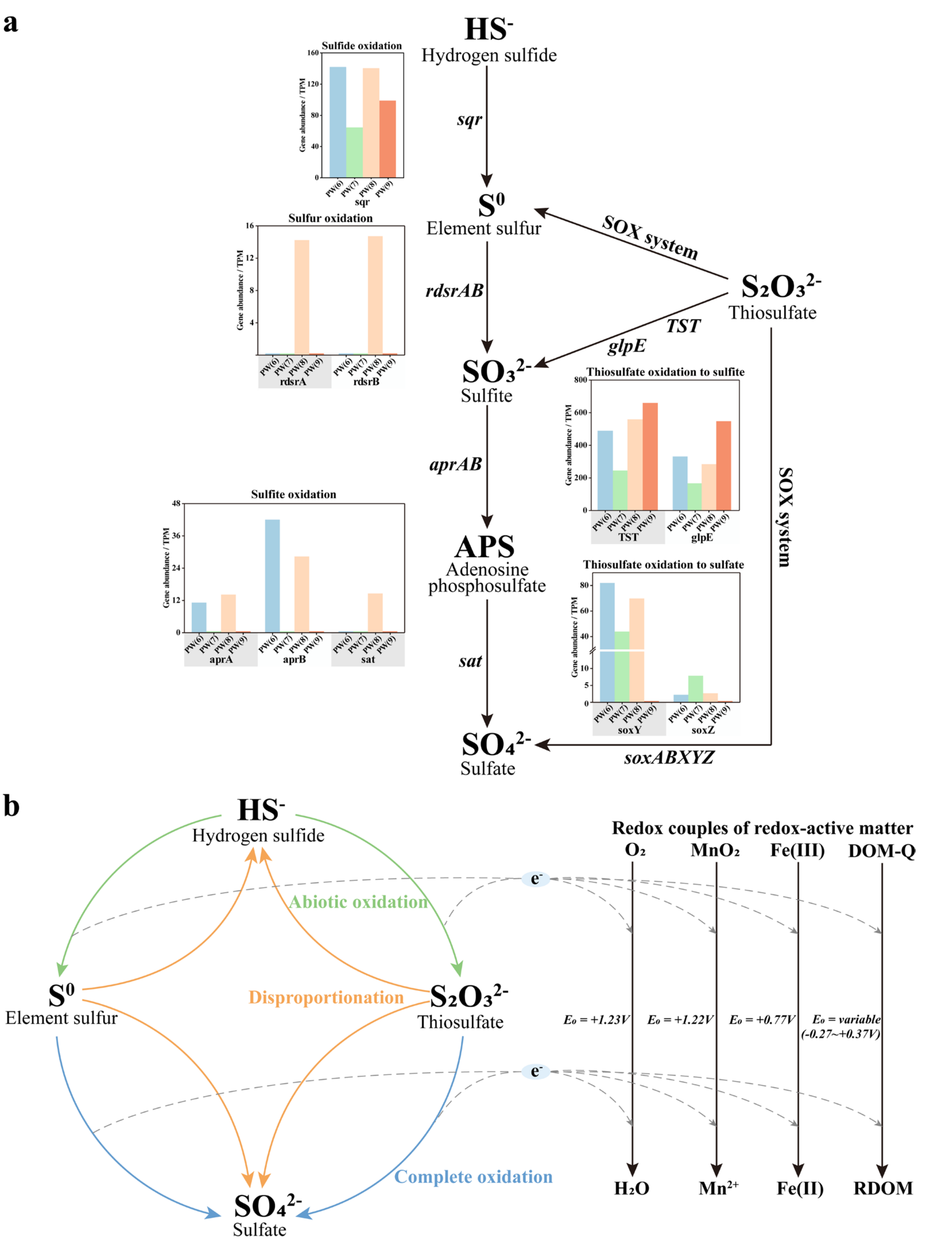

Supplementary Fig. S15. Hidden sulfur oxidation processes for oxidation of reduced sulfur compounds to sulfate. (a) Microbial metabolic pathways for oxidation of sulfur compounds. Inserted histograms depict the key gene abundance among various stages of PW microbiomes. Sulfur oxidation and sulfate generation were extremely active in the PW microbiomes of August. (b) The abiotic oxidation of reduced sulfur compounds. Redox-active matter such as MnO_2, Fe(III) and quinone moieties of dissolved organic matter (DOM-Q) can mediate the first step of sulfide oxidation to elemental sulfur and thiosulfate. Intermediate sulfur compounds will further undergo either reduction back to sulfide, disproportionation to sulfate and sulfide, or complete oxidation to sulfate in the presence of MnO_2 and Fe(III) with high redox potentials. Standard redox potentials of relevant redox-active matter were at 298.15 K and 1 atm [18, 26].

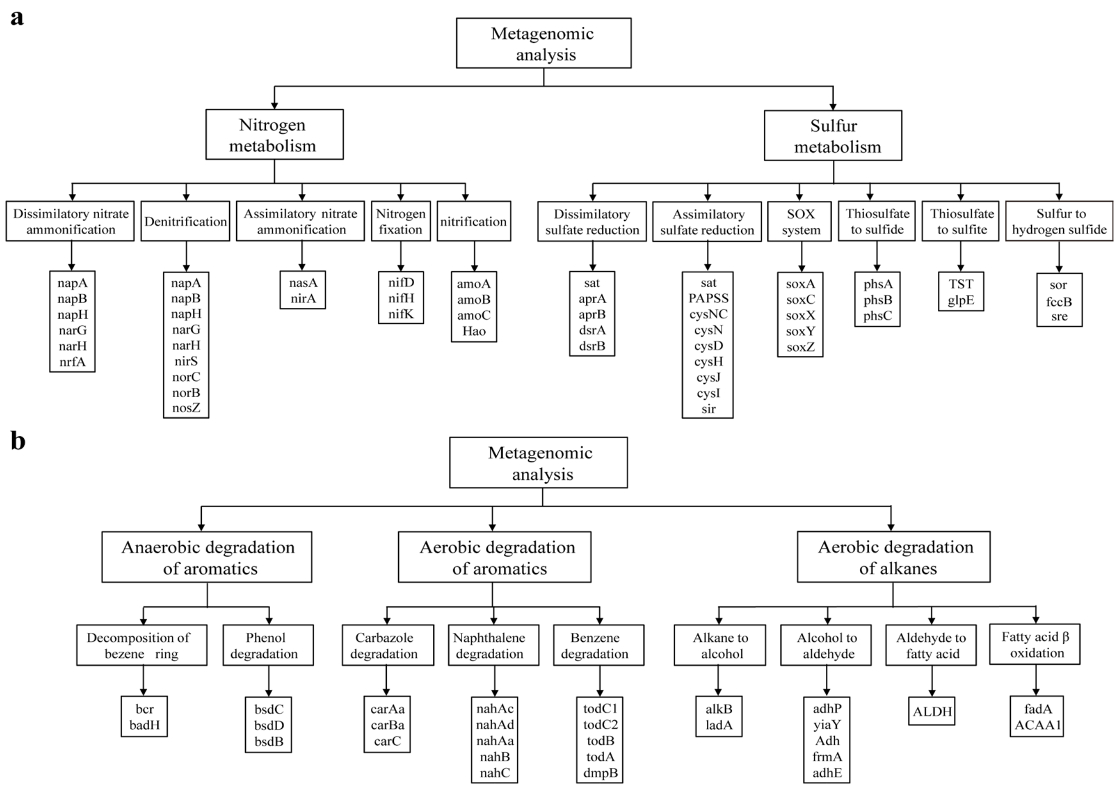

Supplementary Fig. S16. Summary of key genes related to sulfur, nitrogen cycling and hydrocarbon degradation that were investigated in the metagenomics analysis.

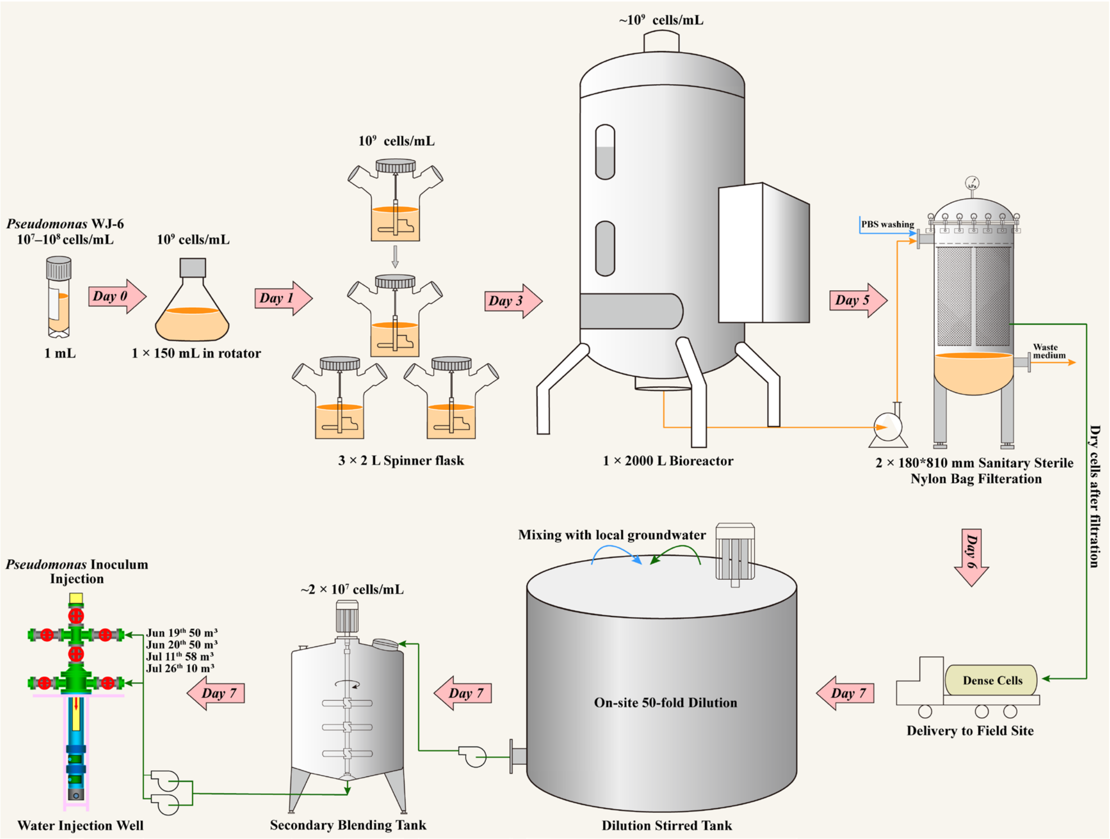

Supplementary Fig. S17. The process flow diagram (PFD) for the preparation of exogenous *Pseudomonas* strain inoculum. The vast volume of *Pseudomonas* solutions was harvested through progressive scale-up fermentation processes in sterile LB culture medium at 35℃. Fermented bacterial liquids were pumped through two 180 * 810 mm Nylon filtration bags with 1-μm pore size at 0.2–0.4 MPa, and cells inside the bags were further washed with 0.01M PBS to remove residual culture medium before being transporting to the field site. The injection inoculum was prepared by mixing dry cells with local clean groundwater in two tandem blending tanks, thereby diluting to a 50-fold volume of the original fermentation liquid.

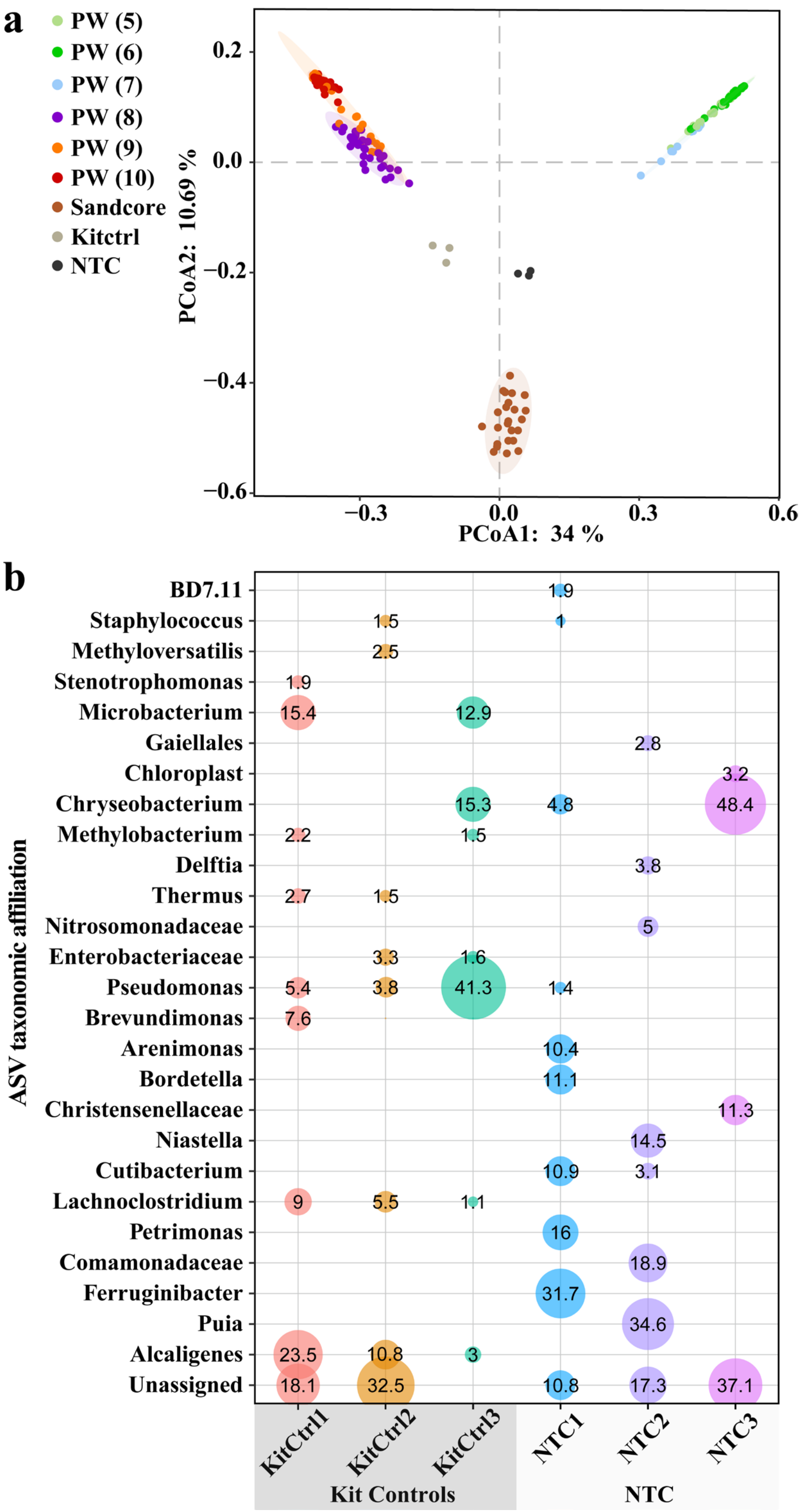

Supplementary Fig. S18. Assessment of potential reagent and laboratory contaminations and effects on biological samples. (a) PCoA analysis based on Bray-Curtis distance. Controls and samples were clearly separated without any overlap, showing the evident taxonomic differences between them and, thus, negligible impacts on our biological samples. (b) The 16S rRNA gene profiles for DNA extraction kit controls (KitCtrl) and no-template controls (NTCs) to identify the contaminant taxa. KitCtrl and NTC were both loaded onto sterile 96-well plate along with biological samples. Only ASVs with relatively abundance larger than 1% are shown.

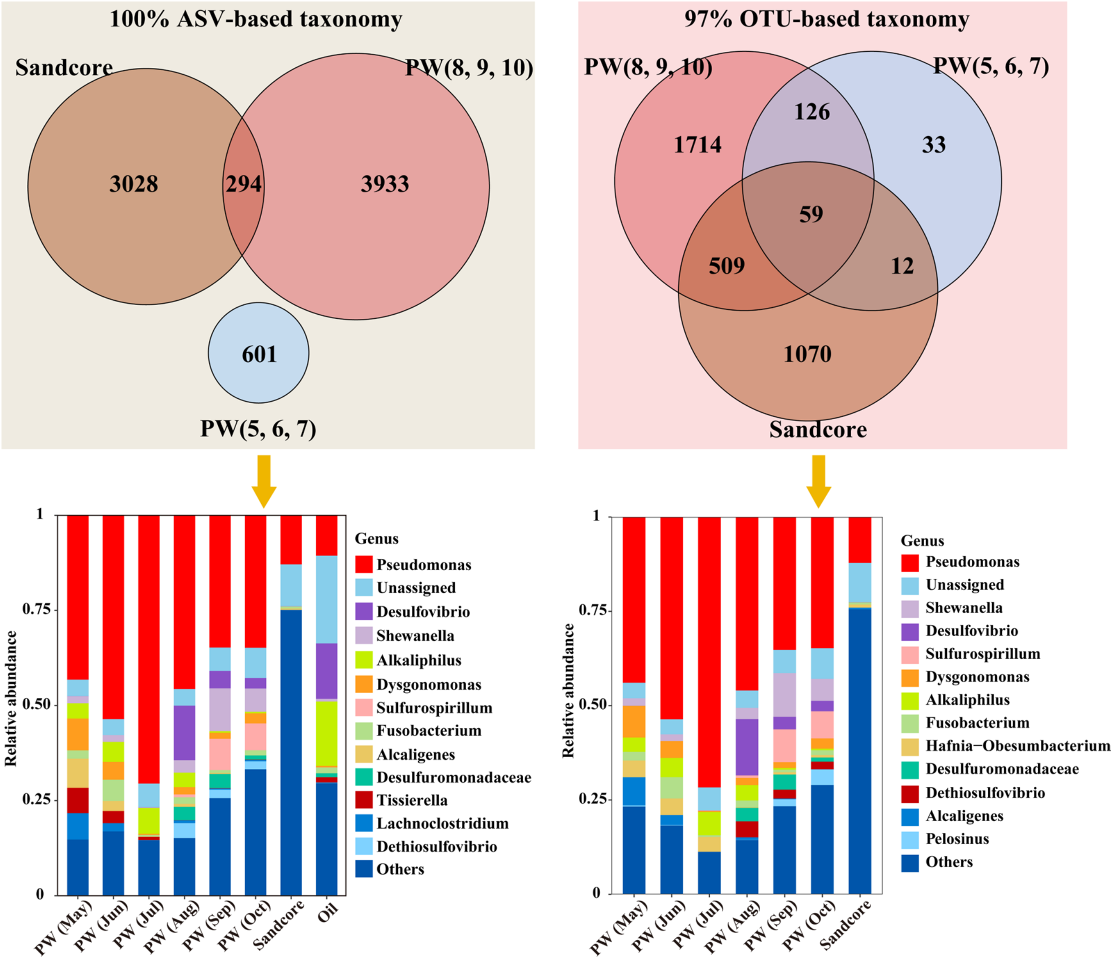

Supplementary Fig. S19. Venn diagram and taxonomic compositions at different taxonomic classification levels. There are a number of shared OTUs (59) among initial PW, post-intervention PW and sandstone cores, but no shared ASVs were found, indicating that the taxonomic classification methods (ASV versus OTU) have little influence on taxonomic compositions but significantly affect our search for the same species. However, the results do not impact our conclusions that many emerging species in PW after the interventions could derive from sandstone cores and oil phases.

Supplementary Tables

Supplementary Table S1. Primers sequences and quantitative reverse transcription PCR (RT-qPCR) programs used in this study.

**Target**	**Gene type**	**Primer set**	**Sequence (5'–3')**	**Expected size**	**PCR conditions**	**Reference**
Bacteria	16S rRNA gene	8F<br>338R	5′-AGA GTT TGA TYM TGG CTC-3′<br>5′-GCT GCC TCC CGT AGG AGT-3′	290 bp	55°C<br>30 cycles	[27]
			N-cycle			
Nitrate reduction (NO_3^- to NO_2^-)	napA gene	napAF1<br>napAR1	5′-CTG GAC NAT GGG YTT NAA CCA-3′<br>5′-CCT TCY TTY TCN ACC CAC AT-3′	492 bp	55°C<br>30 cycles	[28]
Denitrification (N_2O to N_2)	nosZ gene	nosZ-F<br>nosZ1622R	5′-CGY TGT TCM TCG ACA GCC AG-3′<br>5′-CGC RAS GGC AAS AAG GTS CG-3′	410 bp	53°C<br>35 cycles	[29]
			S-cycle			
Sulfate reduction (SO_3^{2-} to S^{2-})	dsrA gene	DSR1F+<br>DSR-R	5′-ACS CAC TGG AAG CAC GGC GG-3′<br>5′-GTG GMR CCG TGC AKR TTG G-3′	221 bp	55°C<br>30 cycles	[30, 31]
Sulfate reduction (SO_3^{2-} to S^{2-})	dsrB gene	DSRp2060F<br>DSR4R	5′-CAA CAT CGT YCA YAC CCA GGG-3′<br>5′-GTG TAG CAG TTA CCG CA-3′	350 bp	55°C<br>30 cycles	[32]

Supplementary Table S2. SARA and viscosity analysis of heavy oil from field trails before and after the exogenous bacteria intervention.

**Sample**	**Saturates, wt%**	**Aromatics, wt%**	**Resins, wt%**	**Asphaltenes, wt%**	**Viscosity at 25°C (mPa·s)**
**Initial heavy oil composition (before May)**	23.74 ± 3.45	15.55 ± 1.34	28.23 ± 5.12	15.31 ± 4.60	302,000
**Oil composition after bacterial modulation (Oct)**	23.09 ± 7.22	8.46 ± 1.67	54.03 ± 8.04	3.88 ± 0.52	3,104

*Note: multiple sample tests, expressed as mean ± SEM. Original data is available in Supplementary Dataset S1.

Supplementary Table S3. Element analysis of heavy oil from field trails before and after the exogenous bacteria intervention.

**Sample**	**S (mg/kg)**	**Fe (mg/kg)**	**Mg (mg/kg)**	**K (mg/kg)**	**Na (mg/kg)**	**Ca (mg/kg)**	**Pb (mg/kg)**	**Cu (mg/kg)**	**Al (mg/kg)**
**Oil (initial reservoirs)**	3,550	15,400	221	330	123	3,110	10,400	22,700	2,650
**Oil (Oct, average)**	2,070	700	<10	<10	<10	909	3,000	3,800	390

Supplementary Table S4. The degree of small-world properties of subnetworks.

Topological indices	PW (5,6,7)	PW (8,9,10)	Sandstone core
CC	0.7727	0.685	0.849
CC (random)	0.011565	0.057745	0.019074
APL	1.525	2.758	7.286
APL (random)	5.036881	2.461317	2.725491
**σ (small world)**	**220.6791**	**10.58647**	**16.65063**

*CC, clustering coefficient; APL, average path length; σ, small-world coefficient, $\sigma = \frac{CC/CC_{random}}{APL/APL_{random}}$; the categorical definition of small-world network implies $\sigma > 1$ [33]; CC (random) and APL (random) indicates the topological properties of 1000 Erdős–Rényi random networks established with the corresponding number of nodes and edges.

Supplementary Table S5. The physical characteristics and bacterial cell densities of various petroleum reservoir habitats.

Description	PW	Sandstone core
**Water content**	–	$4.52 \pm 0.75\%$
**Oil content**	–	$10.93 \pm 0.77\%$
**Cell densities by qPCR**	$(6.31 \pm 0.81) \times 10^6$ CFU/mL	–
**Culturable aerobes**	$(2.13 \pm 0.40) \times 10^5$ CFU/mL	$(9.39 \pm 3.05) \times 10^6$ CFU/g dry weight
**Culturable anaerobes**	$(1.76 \pm 0.88) \times 10^4$ CFU/mL	$(5.14 \pm 1.65) \times 10^5$ CFU/g dry weight

*Note: CFU, Colony Forming Units; multiple sample tests, expressed as mean ± SEM. Original data is available in Supplementary Dataset S1. The missing of qPCR results for core samples since all DNA samples were run out during high-throughput sequencing.

References

1. Xia W, Du Z, Cui Q, Dong H, Wang F, He P, et al. Biosurfactant produced by novel Pseudomonas sp. WJ6 with biodegradation of n-alkanes and polycyclic aromatic hydrocarbons. *J Hazard Mater* 2014; 276: 489–498.

2. Xia W, Tong L, Jin T, Hu C, Zhang L, Shi L, et al. N,S-Heterocycles biodegradation and biosurfactantproduction under CO2/N2 conditions by Pseudomonas and its application on heavy oil recovery. *Chem Eng J* 2021; 413: 128771.

3. Li L, Shen X, Zhao C, Liu Q, Liu X, Wu Y. Biodegradation of dibenzothiophene by efficient Pseudomonas sp. LKY-5 with the production of a biosurfactant. *Ecotoxicol Environ Saf* 2019; 176: 50–57.

4. Martínez I, Mohamed ME-S, García JL, Díaz E. Enhancing biodesulfurization by engineering a synthetic dibenzothiophene mineralization pathway. *Front Microbiol* 2022; 13.

5. Maeda K, Nojiri H, Shintani M, Yoshida T, Habe H, Omori T. Complete Nucleotide Sequence of Carbazole/Dioxin-degrading Plasmid pCAR1 in Pseudomonas resinovorans Strain CA10 Indicates its Mosaicity and the Presence of Large Catabolic Transposon Tn4676. *J Mol Biol* 2003; 326: 21–33.

6. Martínez I, El-Said Mohamed M, Santos VE, García JL, García-Ochoa F, Díaz E. Metabolic and process engineering for biodesulfurization in Gram-negative bacteria. *J Biotechnol* 2017; 262: 47–55.

7. Kilbane JJ. Microbial biocatalyst developments to upgrade fossil fuels. *Curr Opin Biotechnol* 2006; 17: 305–314.

8. Engel K, Coyotzi S, Vachon MA, McKelvie JR, Neufeld JD. Validating DNA Extraction Protocols for Bentonite Clay. *mSphere* 2019; 4: e00334-19.

9. Stroes-Gascoyne S, Hamon CJ, Maak P, Russell S. The effects of the physical properties of highly compacted smectitic clay (bentonite) on the culturability of indigenous microorganisms. *Appl Clay Sci* 2010; 47: 155–162.

10. Zhang Z, Qu Y, Li S, Feng K, Wang S, Cai W, et al. Soil bacterial quantification approaches coupling with relative abundances reflecting the changes of taxa. *Sci Rep* 2017; 7: 4837.

11. Frossard A, Hammes F, Gessner MO. Flow Cytometric Assessment of Bacterial Abundance in Soils, Sediments and Sludge. *Front Microbiol* 2016; 7: 903.

12. Jalique DR, Stroes-Gascoyne S, Hamon CJ, Priyanto DG, Kohle C, Evenden WG, et al. Culturability and diversity of microorganisms recovered from an eight-year old highly-compacted, saturated MX-80 Wyoming bentonite plug. *Appl Clay Sci* 2016; 126: 245–250.

13. Grigoryan AA, Jalique DR, Medihala P, Stroes-Gascoyne S, Wolfaardt GM, McKelvie J, et al. Bacterial diversity and production of sulfide in microcosms containing uncompacted

bentonites. *Heliyon* 2018; 4: e00722.

14. Vigneron A, Alsop EB, Lomans BP, Kyrpides NC, Head IM, Tsesmetzis N. Succession in the petroleum reservoir microbiome through an oil field production lifecycle. *ISME J* 2017; 11: 2141–2154.

15. Anantharaman K, Breier JA, Sheik CS, Dick GJ. Evidence for hydrogen oxidation and metabolic plasticity in widespread deep-sea sulfur-oxidizing bacteria. *Proc Natl Acad Sci* 2013; 110: 330–335.

16. Hansel CM, Lentini CJ, Tang Y, Johnston DT, Wankel SD, Jardine PM. Dominance of sulfur-fueled iron oxide reduction in low-sulfate freshwater sediments. *ISME J* 2015; 9: 2400–2412.

17. Heitmann T, Blodau C. Oxidation and incorporation of hydrogen sulfide by dissolved organic matter. *Chem Geol* 2006; 235: 12–20.

18. Aeschbacher M, Sander M, Schwarzenbach RP. Novel Electrochemical Approach to Assess the Redox Properties of Humic Substances. *Environ Sci Technol* 2010; 44: 87–93.

19. Pester M, Knorr K-H, Friedrich M, Wagner M, Loy A. Sulfate-reducing microorganisms in wetlands – fameless actors in carbon cycling and climate change. *Front Microbiol* 2012; 3.

20. Beman JM, Bertics V, Braunschweiler T, Wilson J. Quantification of ammonia oxidation rates and the distribution of ammonia-oxidizing Archaea and Bacteria in marine sediment depth profiles from Catalina Island, California. *Front Microbiol* 2012; 3.

21. Francis CA, Beman JM, Kuypers MMM. New processes and players in the nitrogen cycle: the microbial ecology of anaerobic and archaeal ammonia oxidation. *ISME J* 2007; 1: 19–27.

22. Huang S, Chen C, Peng X, Jaffé PR. Environmental factors affecting the presence of Acidimicrobiaceae and ammonium removal under iron-reducing conditions in soil environments. *Soil Biol Biochem* 2016; 98: 148–158.

23. Huang S, Jaffé PR. Isolation and characterization of an ammonium-oxidizing iron reducer: Acidimicrobiaceae sp. A6. *PLOS ONE* 2018; 13: e0194007.

24. Sawayama S. Possibility of anoxic ferric ammonium oxidation. *J Biosci Bioeng* 2006; 101: 70–72.

25. Eisenhofer R, Minich JJ, Marotz C, Cooper A, Knight R, Weyrich LS. Contamination in Low Microbial Biomass Microbiome Studies: Issues and Recommendations. *Trends Microbiol* 2019; 27: 105–117.

26. Bard AJ, Parsons R, Jordan J. Standard potentials in aqueous solution. 1985.

27. Gao P, Li G, Li Y, Li Y, Tian H, Wang Y, et al. An Exogenous Surfactant-Producing Bacillus subtilis Facilitates Indigenous Microbial Enhanced Oil Recovery. *Front Microbiol* 2016; **7**.

28. Feng W-W, Liu J-F, Gu J-D, Mu B-Z. Nitrate-reducing community in production water of three oil reservoirs and their responses to different carbon sources revealed by nitrate-

reductase encoding gene (napA). *Int Biodeterior Biodegrad* 2011; **65**: 1081–1086.

29. Throbäck IN, Enwall K, Jarvis Å, Hallin S. Reassessing PCR primers targeting nirS, nirK and nosZ genes for community surveys of denitrifying bacteria with DGGE. *FEMS Microbiol Ecol* 2004; **49**: 401–417.

30. Gao H, Wang C, Chen J, Wang P, Zhang J, Zhang B, et al. Enhancement effects of decabromodiphenyl ether on microbial sulfate reduction in eutrophic lake sediments: A study on sulfate-reducing bacteria using dsrA and dsrB amplicon sequencing. *Sci Total Environ* 2022; **843**: 157073.

31. Kondo R, Nedwell DB, Purdy KJ, Silva SQ. Detection and Enumeration of Sulphate-Reducing Bacteria in Estuarine Sediments by Competitive PCR. *Geomicrobiol J* 2004; **21**: 145–157.

32. Geets J, Borremans B, Diels L, Springael D, Vangronsveld J, van der Lelie D, et al. DsrB gene-based DGGE for community and diversity surveys of sulfate-reducing bacteria. *J Microbiol Methods* 2006; **66**: 194–205.

33. Humphries MD, Gurney K. Network 'Small-World-Ness': A Quantitative Method for Determining Canonical Network Equivalence. *PLOS ONE* 2008; 3: e0002051.
